# Decreased spliceosome fidelity and *egl-8* intron retention inhibit mTORC1 signaling to promote longevity

**DOI:** 10.1038/s43587-022-00275-z

**Published:** 2022-09-19

**Authors:** Wenming Huang, Chun Kew, Stephanie de Alcantara Fernandes, Anna Löhrke, Lynn Han, Constantinos Demetriades, Adam Antebi

**Affiliations:** 1 Max Planck Institute for Biology of Ageing, Cologne, Germany; 2 Cologne Graduate School for Aging Research, Cologne, Germany; 3 Cologne Excellence Cluster on Cellular Stress Responses in Aging-Associated Diseases, University of Cologne, Cologne, Germany; 4 Present Address: Institute of Biochemistry II, Goethe-Universität, Frankfurt am Main, Germany; 5 Present Address: Weill Medical College of Cornell University, New York, NY USA

**Keywords:** RNA splicing, RNA splicing, Cell signalling, Ageing

## Abstract

Changes in splicing fidelity are associated with loss of homeostasis and aging, yet only a handful of splicing factors have been shown to be causally required to promote longevity, and the underlying mechanisms and downstream targets in these paradigms remain elusive. Surprisingly, we found a hypomorphic mutation within ribonucleoprotein RNP-6/poly(U)-binding factor 60 kDa (PUF60), a spliceosome component promoting weak 3′-splice site recognition, which causes aberrant splicing, elevates stress responses and enhances longevity in *Caenorhabditis elegans*. Through genetic suppressor screens, we identify a gain-of-function mutation within *rbm-39*, an RNP-6-interacting splicing factor, which increases nuclear speckle formation, alleviates splicing defects and curtails longevity caused by *rnp-6* mutation. By leveraging the splicing changes induced by RNP-6/RBM-39 activities, we uncover intron retention in *egl-8*/phospholipase C β4 (PLCB4) as a key splicing target prolonging life. Genetic and biochemical evidence show that neuronal RNP-6/EGL-8 downregulates mammalian target of rapamycin complex 1 (mTORC1) signaling to control organismal lifespan. In mammalian cells, PUF60 downregulation also potently and specifically inhibits mTORC1 signaling. Altogether, our results reveal that splicing fidelity modulates lifespan through mTOR signaling.

## Abstract (web summary)

RNA splicing has a role in aging and longevity, but the mechanisms involved are incompletely understood. Here the authors show that mRNA splicing components, RNP-6 and RBM-39, act in concert to regulate intron retention, inhibit mTORC1 signaling and prolong life in *Caenorhabditis elegans*.

## Main

Pre-messenger RNA splicing is a major step of gene regulation that contributes to proteomic diversity in eukaryotes. In this process, multiple splicing factors and RNAs come together in an organized stepwise fashion to form the spliceosome, acting to remove introns and create alternative mRNAs. Ensuring splicing fidelity and isoform diversity is essential to proper growth, development and homeostasis^1^. Accordingly, mutations in core spliceosomal components that disrupt the splicing process can cause various pathologies, including muscular dystrophies, cancer and neurodegenerative and cardiovascular diseases^2,3^. Splicing has also been recently implicated in playing a role in the aging process located downstream of pro-longevity interventions such as diet restriction and mTOR inhibition^4,5^. However, the relationship between splicing and aging is still unclear and the detailed mechanisms of how the splicing machinery/splicing factors regulate longevity remain largely elusive.

PUF60 encodes an essential splicing factor that binds polyuridine (U) tracts and promotes association of the U2 small nuclear RNP complex (U2 snRNP) with primary transcripts^6,7^. PUF60 is required for cell viability, proliferation and migration in vitro. Its deficiency in humans causes developmental defects^8–10^ and overexpression is associated with tumorigenesis^11,12^, but a role in metabolism and aging is completely unknown. In a previous genetic screen for *C. elegans* longevity regulators using cold tolerance as proxy, we had identified a new mutation in the worm ortholog of PUF60, *rnp-6*, carrying a Gly281Asp substitution (referred to as *rnp-6(G281D)* hereafter) in the second RNA recognition motif (RRM), which alters resistance to multiple abiotic stresses and extends lifespan^13^.

In the present study, to decipher the molecular and cellular mechanisms underlying *rnp-6*-induced physiology, we combined forward genetic screening, transcriptomic profiling and genetic epistasis analysis, and discovered that a RNP-6/RNA-binding protein 39 (RBM-39) spliceosome complex regulates longevity by modulating intron retention of the splicing target *egl-8*/PLCB4. Furthermore, we found that the RNP-6/EGL-8 axis acts mainly within the nervous system and downregulates mTORC1 signaling to confer longevity. In human cells, PUF60 knockdown leads to downregulation of mTORC1 signaling and re-localization of mTOR away from lysosomes, indicating its integral, evolutionarily conserved role in this pathway. Together, our results reveal a new mechanism by which the spliceosome complex regulates lifespan in a multicellular organism, partially through intron retention, and pinpoints potential targets to promote healthy aging.

## Results

### *Rnp-6(G281D)* is a selective reduction-of-function mutation

**Fig. 1:**
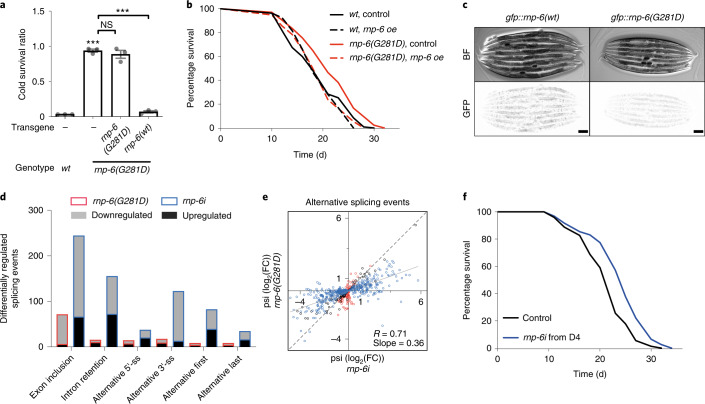
Reduction of *rnp-6* function promotes longevity. **a**, Effect of transgenic *rnp-6* overexpression on rescue of cold tolerance (*n* = 3 independent biological replicates). Mean ± s.e.m.; ^***^P < 0.001 by one-way ANOVA. NS, not significant. **b**, Representative survival plot showing the effect of transgenic *rnp-6* overexpression on rescue of lifespan (*n* = 3). For all lifespan experiments, survival curves depict one representative experiment. The number of worms used and the statistical analysis in each repeat are shown in Supplementary Table 16. **c**, Expression of GFP-tagged endogenous wild-type RNP-6 (*rnp-6(syb645)*) and mutated RNP-6 (*rnp-6(syb626)*) in young adult-stage worms. Scale bars, 100 μm. Top and bottom panels represent bright field (BF) and GFP fluorescent images, respectively. Fluorescence is inverted to show better contrast (*n* = 3). For all imaging experiments, images from a representative experiment are shown. **d**, Quantification of differentially regulated, alternative splicing changes found in *rnp-6(G281D)* and *rnp-6i* (*n* = 3). ss, splice site. **e**, Correlation of *rnp-6(G281D)*- and *rnp-6i*-induced alternative splicing changes. Each dot represents the percentage spliced-in (psi) log_2_(transformed fold-change) (log_2_(FC)) of an event relative to the wild-type control. Blue, red and gray dots indicate the events that are significantly changed by *rnp-6i*, *rnp-6(G281D)* and both, respectively. **f**, Representative survival plot of *rnp-6i* treatment from day 4 of adulthood (*n* = 4). Source data

**Extended Data Fig. 1:**
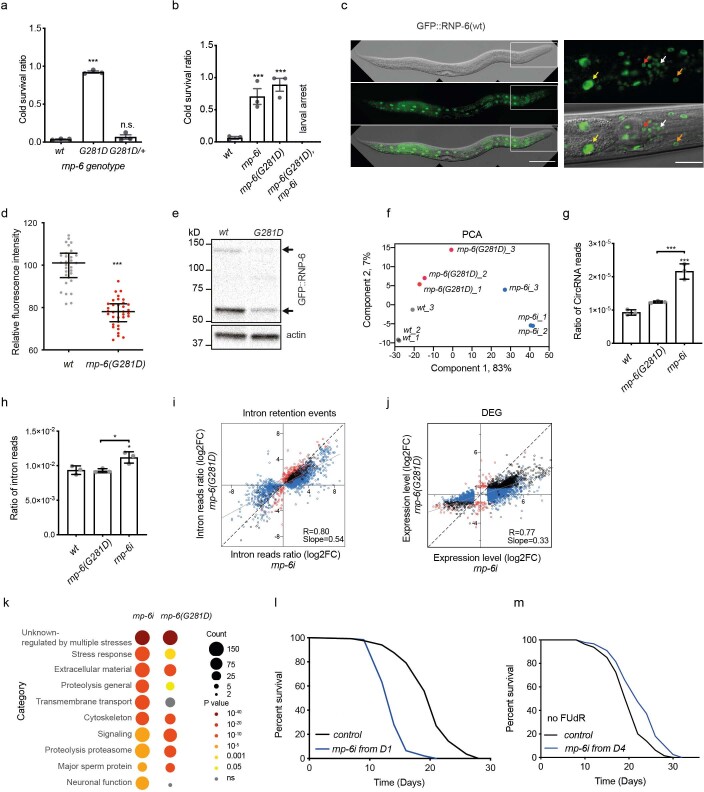
Characterization of *rnp-6(G281D)* mutant. a, Effect of *rnp-6(G281D)* heterozygosity on cold tolerance. n = 3 independent biological repeats. Mean ± SEM. ***P < 0.001; n.s., not significant. one-way ANOVA with Dunnett adjustment. b, Effect of *rnp-6i* on cold tolerance. n = 3. Mean ± SEM. ***P < 0.001. one-way ANOVA with Dunnett adjustment. c, Expression and localization of GFP-tagged RNP-6. RNP-6 localizes in multiple tissues, including gut (yellow arrow), neuron (white arrow), hypodermal cell (green arrow) and pharyngeal muscle cell(red arrow). Scale bar: left panels 100 μm, right panels 20 μm. d, *rnp-6(G281D)* mutation decreases RNP-6 expression level. Quantification of Fig. 1c. n = 3. Mean ± SD. ***P < 0.001. Two-tailed unpaired t-test. e, Western blot of RNP-6. Arrows indicate two isoforms of RNP-6. n = 3. f, Principal components analysis (PCA) of RNAseq dataset for *rnp-6i* and *rnp-6(G281D)*. g-h, Effect of *rnp-6i* and *rnp-6(G281D)* on circularRNA (CircRNA) formation (g) and intron retention (h). n = 3. Mean ±SEM. *P < 0.05; ***P < 0.001. one-way ANOVA with Tukey adjustment. i-j, Correlation of intron retention events and differentially expressed genes (DEG) caused by *rnp-6i* and *rnp-6(G281D)*. Each dot represents the log2-transformed fold changes of an event relative to *wild-type* control. Blue, red and grey dots indicate the events which are significant changed by *rnp-6i*, *rnp-6(G281D)* and both, respectively. k, Functional enrichment of differentially expressed genes in *rnp-6i* and *rnp-6(G281D)*. P-values are calculated from Fisher’s exact tests and adjusted with Bonferroni multiple hypothesis test. P < 0.05 was defined as significant. l, Representative survival plot *rnp-6i* treatment from day 1 adult stage. n = 5. For all lifespan experiments, survival curves depict one representative experiment. The number of worms used in each repeat and the statistical analysis are shown in Supplementary Table S16. m, Representative survival plot of *rnp-6i* treatment from day 4 adult stage without FUDR. n = 3. In panel d, e, i, and m, the results from a representative experiment are shown. Source data

**Extended Data Fig. 2:**
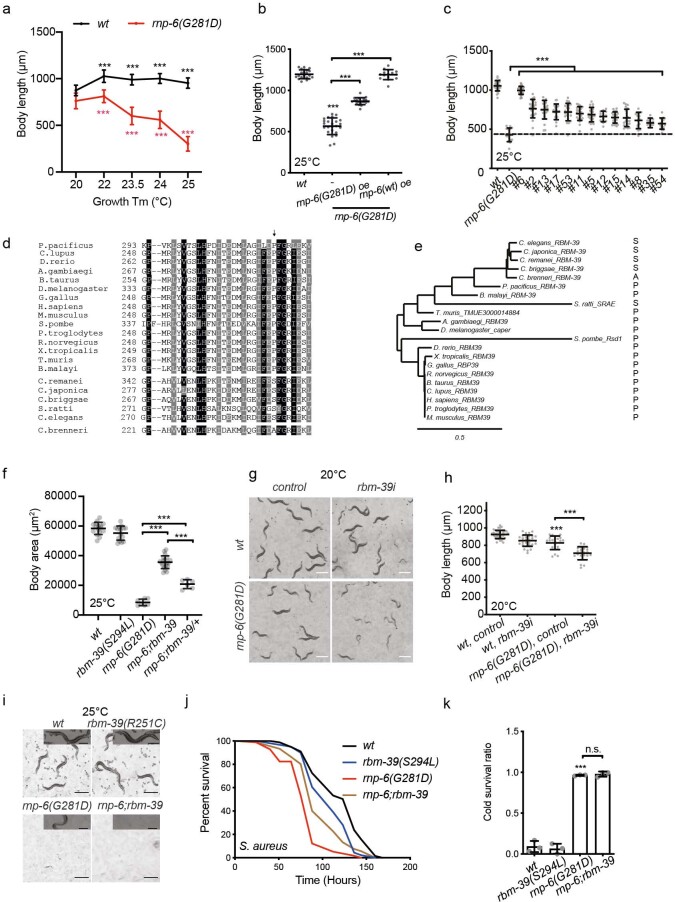
Genetic screen reveals suppressors of *rnp-6(G281D)*. a, The effects of growth temperature on *rnp-6(G281D)* development. N = 13-53 worms for each condition. Mean ± SD. ***P < 0.001, One-way ANOVA with Dunnett adjustment. n = 2 biological repeats. Black and red asterisks represent the comparison within *wt* and *rnp-6(G281D)* groups, respectively. b, Effect of *rnp-6* overexpression on temperature sensitive phenotype. n = 3. Mean ± SD. ***P < 0.001. one-way ANOVA with Dunnett adjustment. c, Summary of the mutants which suppress *rnp-6(G281D) ts* phenotype. Mutants #6 and #2 correspond to *dh1187* mutant and *dh1183* mutant, respectively. N = 12-29 worms for each condition. Mean ± SD. ***P < 0.001. one-way ANOVA with Dunnett adjustment. n = 2 biological repeats. d-e, Alignment and Phylogenetic analysis of RBM-39 with its orthologs from different species. Arrow head indicates amino acid S294 of *C. elegans*. f, Effect of *rbm-39(S294L)* heterozygosity on *ts* phenotype. n = 3. Mean ± SD. ***P < 0.001, One-way ANOVA. g-h, Effect of *rbm-39i* on *rnp-6(G281D)* growth under standard (20 °C) growth temperature. Scale bar, 500 μm. n = 3. Mean ± SD. ***P < 0.001. one-way ANOVA with Tukey adjustment. i, Effect of *rbm-39* reduction-of-function on *rnp-6(G281D) ts* phenotype. n = 3. Scale bar, 500 μm. In the magnified insets images, scale bar, 200 μm. j, Representative survival plot of *rbm-39(S294L)* upon *S. aureus* infection. n = 3. For all the infection experiments, survival curves depict one representative experiment. Other repeats are shown in Supplementary Table 17. k, Effect of *rbm-39(S294L)* on cold tolerance. n = 3. Mean ± SD. ***P < 0.001. one-way ANOVA with Dunnett adjustment. In panels a-c and f-k, data from a representative experiment are shown. Source data

We first sought to characterize the nature of *rnp-6(G281D)* in more detail and found that this mutation behaved as a recessive, hypomorphic allele, because: (1) *rnp-6(G281D)*/+ heterozygotes were as cold sensitive as wild-type (*wt* or WT) controls (Extended Data Fig. 1a); (2) knockdown of *rnp-6* by RNAi bacterial feeding (*rnp-6i*) enhanced cold tolerance in the wild-type background and caused developmental arrest in *rnp-6(G281D)* mutants (Extended Data Fig. 1b); and (3) overexpression of *rnp-6(wt)* but not a *rnp-6(G281D)* transgene fully rescued the *rnp-6(G281D)* cold tolerance phenotype (Fig. 1a). Moreover, the *rnp-6(wt)* transgene also fully rescued the longevity phenotype (Fig. 1b). To characterize the cellular function of *rnp-6*, we tagged endogenous *rnp-6* with green fluorescent protein (GFP) using clustered regularly interspaced short palindromic repeats (CRISPR)–Cas9. Consistent with its role as an essential splicing factor, *rnp-6(wt)* was ubiquitously expressed in all examined tissues and mainly localized in the nucleus (Extended Data Fig. 1c). GFP-tagged endogenous *rnp-6(G281D)* showed a similar expression pattern, but was present at significantly lower levels (Fig. 1c and Extended Data Fig. 1d), and further validated by western blotting (Extended Data Fig. 1e), suggesting a reduction of function. RNA-sequencing (RNA-seq) analysis also showed that *rnp-6(G281D)* caused changes in mRNA processing and transcription similar to, but not as extensive as, *rnp-6i*, including alternative splicing, intron retention and circular RNA formation (Fig. 1d,e, Extended Data Fig. 1f–i and Supplementary Tables 1 and 3), as well as differential gene expression (Extended Data Fig. 1j and Supplementary Table 4), confirming that *rnp-6(G281D)* represents a reduction-of-function mutation. Notably, ~80% of the differentially expressed genes (DEGs) (1,142 of 1,366 genes in *rnp-6(G281D)* and 3,730 of 4,707 genes in *rnp-6i*) were upregulated (Supplementary Table 4). Gene ontology (GO) analysis of the DEGs showed that stress response was among the most enriched physiological categories in both *rnp-6(G281D)* and *rnp-6i* (Extended Data Fig. 1k and Supplementary Table 5), revealing that impaired spliceosome function triggers cellular stress responses.

Next, we asked whether *rnp-6i* mimicked *rnp-6(G281D)* longevity. To bypass developmental defects, we performed *rnp-6i* during adulthood. Whereas *rnp-6i* from day 1 adults (coinciding with the onset of reproduction) decreased the lifespan (Extended Data Fig. 1l), *rnp-6i* initiated from day 4 adults (coinciding near the end of reproduction) onward significantly extended it (Fig. 1f). This happens in either the presence or the absence of fluoro-2′-deoxyuridine-5′-phosphate (FUDR; an inhibitor of DNA synthesis commonly used in *C. elegans* lifespan experiments to prevent eggs from hatching), suggesting that lifespan extension is independent of progeny production (Extended Data Fig. 1m). These results reveal that, like several other essential genes^14,15^, *rnp-6* shows antagonistic pleiotropy; knockdown can be detrimental early in life but beneficial later and imply that the fine-tuning of *rnp-6* activity is critical for longevity.

### *Rbm-39(S294L)* ameliorates *rnp-6(G281D)* defects and suppresses longevity

**Fig. 2:**
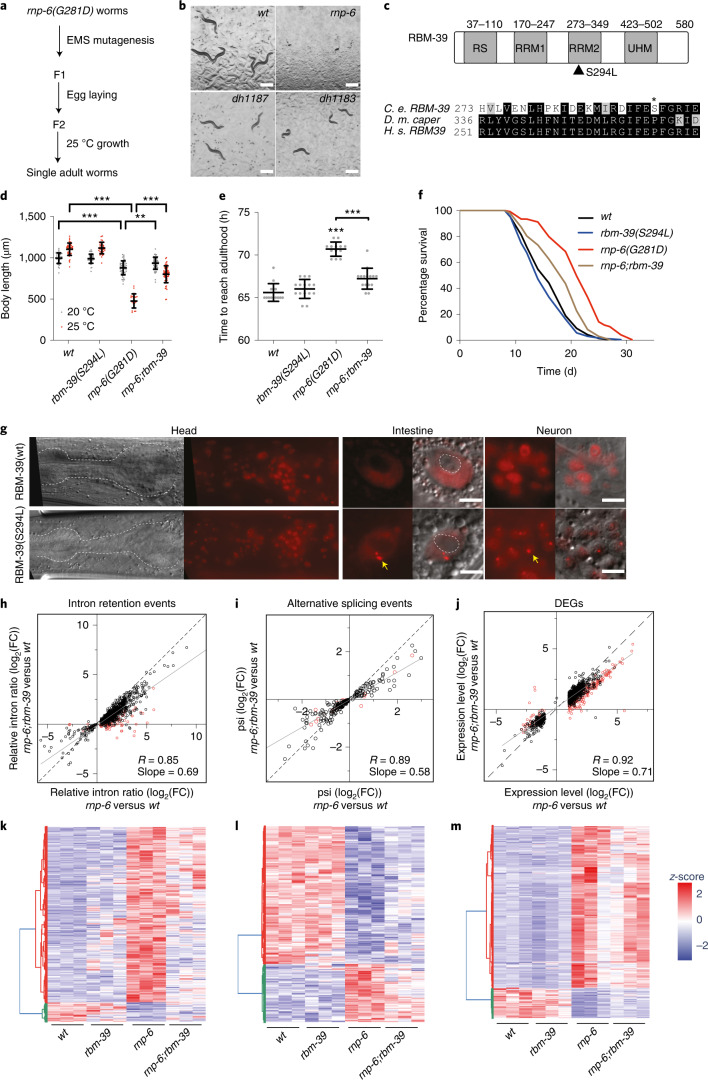
*Rbm-39* functionally interacts with *rnp-6*. **a**, Schematic workflow of the suppressor screen. **b**, Representative images of *dh1183* and *dh1187* suppressors grown at the restrictive temperature of 25 °C. Scale bars, 500 μm. **c**, Protein domain structure of RBM-39 and sequence alignment of RBM-39 homologs in the vicinity of the mutation site. Filled triangle and asterisk denote the location of the serine 294-to-leucine mutation. *C. e*., *C. elegans*; *D. m*., *D. melanogaster*; *H. s*., *H. sapiens*. **d**–**f**, Effect of *rbm-39(S294L)* on *rnp-6(G281D)* body length (**d**), developmental rate (**e**) and longevity (**f**) phenotype (*n* = 3). Mean ± s.d. shown in **d** and **e**. Data from a representative experiment are shown. ^***^P < 0.001, by one-way ANOVA. **g**, Nuclear localization of mKate2-tagged RBM-39. The dotted lines in the head and intestine indicate the boundary of the pharynx and nucleolus, respectively. The yellow arrows indicate RBM-39(Ser294Leu) intranuclear puncta. Scale bars, 5 μm. **h**–**m**, Effect of *rbm-39(S294L)* on intron retention (**h** and **k**, 954 events), alternative splicing (**i** and **l**, 251 events) and gene expression changes (**j** and **m**, 1,709 events) as shown by scatter plots and *z*-score heat maps. Each dot represents the log_2_(FC) of an event relative to the wild-type control. Red dots denote the events that are significantly suppressed by *rbm-39(S294L)*. psi, percentage spliced-in. Source data are shown in Supplementary Tables 7–9. Source data

To dissect the functional network underlying *rnp-6* longevity, we performed unbiased genetic suppressor screens. Notably, we observed that *rnp-6(G281D)* exhibited a tight temperature-sensitive (*ts*) growth phenotype, which could be fully rescued by *rnp-6(wt)* overexpression (Extended Data Fig. 2a,b). We reasoned that those crucial regulators that suppress the *ts* defect could also alleviate other *rnp-6(G281D)* phenotypes. We screened ~20,000 genomes and isolated 13 mutants (Fig. 2a and Extended Data Fig. 2c). By combining Hawaiian single-nucleotide polymorphism (SNP) variant mapping, whole-genome sequencing and CRISPR–Cas9 gene editing, we succeeded in identifying two candidates: *rnp-6(dh1187)* and *rbm-39(dh1183)* (Fig. 2b). The *rnp-6(dh1187)* intragenic mutation led to a glutamate-to-lysine substitution (*rnp-6(E161K)*), which corresponds to Glu188 in human PUF60 (Extended Data Fig. 4a,b). This residue mediates interdomain RRM1–RRM2 contacts in the PUF60 crystal structure^16^ and may affect salt bridge formation. *Rbm-39* encodes an RNA-binding protein, the human ortholog of which, RBM39, functions as a splicing factor and is involved in early spliceosome assembly^17^. Similar to PUF60, RBM39 contains two central RRM domains and a carboxy-terminal U2AF-homology motif (UHM) domain, but additionally harbors an amino-terminal arginine–serine-rich (RS) domain (Fig. 2c) implicated in nuclear speckle formation^18^. The *rbm-39(dh1183)* mutation caused a serine-to-leucine substitution (Ser294Leu) in the second RRM (Fig. 2c). This residue is conserved in nematodes, but changed to proline in higher organisms (Extended Data Fig. 2d,e). It is interesting that a proline-to-serine substitution at this same position in human RBM39 changes its conformation and renders resistance to the anti-cancer drug indisulam, an aryl sulfonamide that facilitates RBM39 proteasomal degradation^19^, highlighting the pivotal role of this residue in regulating RBM39 function.

*Rbm-39(S294L)* represents a semidominant allele because *rbm-39(S294L)/*+ heterozygotes partially suppressed the *rnp-6(G281D) ts* phenotype (Extended Data Fig. 2f). To clarify *rbm-39* function further, we tested the effect of decreased *rbm-39* activity on *rnp-6(G281D) ts* phenotypes. Unlike *rbm-39(S294L), rbm-39* RNA interference (RNAi) knockdown (*rbm-39i*) generally exacerbated *rnp-6(G281D)* phenotypes and caused a further decrease in body size, yet had little effect on wild-type controls (Extended Data Fig. 2g,h). Similarly, a reduction-of-function allele *rbm-39(R251C)*^20^ further delayed developmental rate, decreased body size at 20 °C and caused complete embryonic lethality at 25 °C (Extended Data Fig. 2i). These results suggest that *rbm-39* and *rnp-6* function in concert, and support the notion that the *rbm-39(S294L)* suppressor probably defines a specific change- or gain-of-function allele.

As *rbm-39(S294L)* largely reversed the *ts* defect (Fig. 2d), we next asked whether it also suppressed other *rnp-6(G281D)* phenotypes visible at the permissive temperature (20 °C). Although showing little effect on its own, *rbm-39(S294L*) significantly restored body size (Fig. 2d), developmental rate (Fig. 2e) and infection tolerance (Extended Data Fig. 2j), and diminished *rnp-6(G281D)* lifespan extension (Fig. 2f), suggesting that *rbm-39(S294L*) ameliorates *rnp-6(G281D)* function*. Rbm-39(S294L)* did not affect *rnp-6(G281D)* cold tolerance (Extended Data Fig. 2k), however, suggesting an uncoupling of this phenotype and longevity.

**Extended Data Fig. 3:**
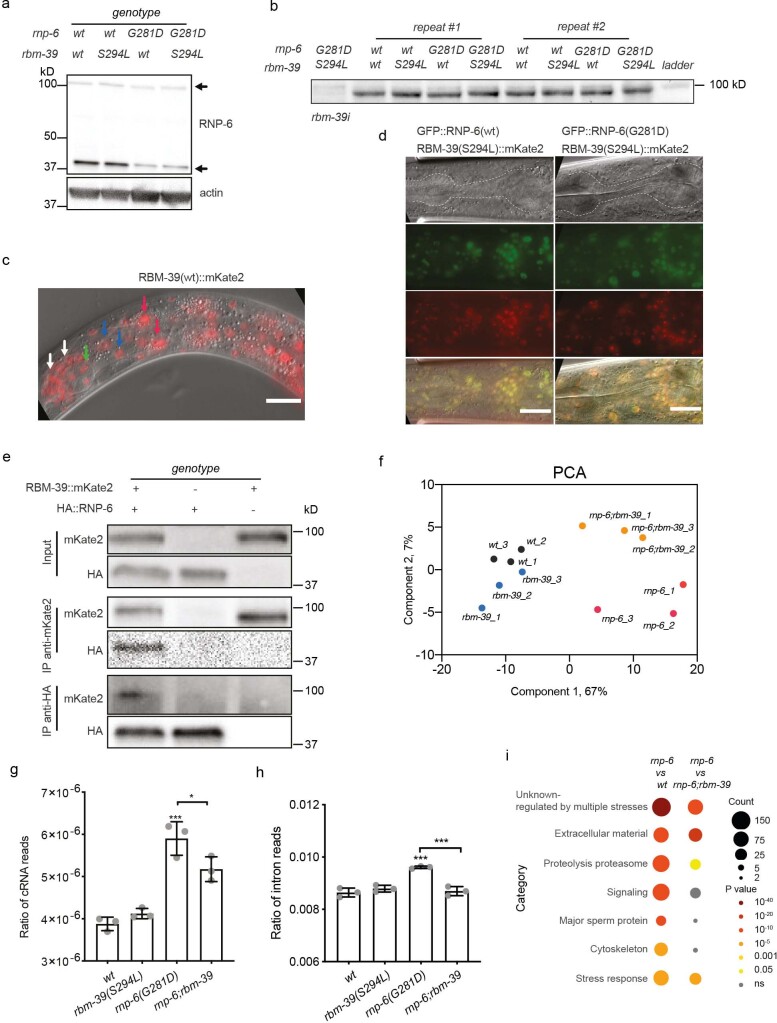
Effect of *rbm-39(S294L)* on *rnp-6(G281D)* function. a, Western blot of RNP-6. n = 2. b, Western blot of RBM-39. n = 2. c, Expression and localization of RBM-39. RBM-39(wt) is ubiquitously expressed in all the checked tissues including neurons (white arrows), muscle (green arrow), gut (red arrows) and hypodermal cells (blue arrow). scale bar, 20 μm. n = 3. d, Localization of RNP-6 and RBM-39. Scale bars, 20 μm. n = 3. e, Co-immunoprecipitation (IP) of RBM-39::mKate2 and HA::RNP-6. Antibodies against mKate2 and HA were used for immunoprecipitation and Western blot. n = 1. “+” and “–” in genotype indicate the protein is tagged and untagged, respectively. f, PCA of RNAseq dataset for *rbm-39(S294L)*. g-h, Effect of *rbm-39(S294L)* on circular RNA formation (g) and intron retention (h). n = 3. Mean ± SEM. *P = 0.0255; ***P < 0.001. one-way ANOVA with Dunnett adjustment. i, Functional enrichment of differentially expressed genes suppressed by *rbm-39(S294L)*. P-values are calculated from Fisher’s exact tests and adjusted with Bonferroni multiple hypothesis test. P < 0.05 was defined as significant. In panels a-d, data from a representative experiment are shown. Source data

To address the potential mechanisms behind *rbm-39(S294L)*-mediated suppression, we first examined whether *rbm-39(S294L)* restored reduced *rnp-6(G281D)* protein levels. Western blotting experiments showed, however, that *rbm-39(S294L)* had no impact on either *rbm-39* or *rnp-6* protein levels (Extended Data Fig. 3a,b). We then wondered whether *rbm-39(S294L)* altered the subcellular localization of RNP-6 or RBM-39. Endogenously mKate2-tagged RBM-39(WT) and RBM-39(Ser294Leu) were ubiquitously expressed and found mainly within the nucleus of various cell types, similar to RNP-6 (Fig. 2g and Extended Data Fig. 3c). It is interesting that we observed that the RBM-39(Ser294Leu) mutant protein, but not the RBM-39(WT) protein, formed prominent discrete puncta within the nucleus without altering RNP-6 localization (Fig. 2g and Extended Data Fig. 3d). These puncta resembled nuclear speckles implicated in regulating transcription and splicing^21,22^. Time-lapse imaging showed that these puncta were, like other nuclear speckles, highly dynamic (Extended Data Video 1). In addition, RBM-39 and RNP-6 mutually co-immunoprecipitated (Extended Data Fig. 3e), suggesting that they associate in a complex. These results imply that *rbm-39(S294L*) might alleviate *rnp-6(G281D)* defects by enhancing splicing activity. To test this hypothesis, we performed RNA-seq analysis with *wt*, *rnp-6(G281D), rbm-39(S294L)* and *rnp-6;rbm-39* double mutants. In accord with our idea, we observed that *rbm-39(S294L)* altered the transcriptional profile (Extended Data Fig. 3f) and decreased total circular RNA and intron reads of the *rnp-6(G281D*) mutant (Extended Data Fig. 3g,h and Supplementary Tables 6 and 7). Furthermore, *rbm-39(S294L)* significantly suppressed intron retention (115 out of 954 events) (Fig. 2h,k and Supplementary Table 7), alternative splicing (19 out of 251 events) (Fig. 2i,l and Supplementary Table 8), and differential gene expression changes (275 out of 1,709 events) (Fig. 2j,m and Supplementary Table 9) caused by *rnp-6(G281D*), and globally trended toward alleviating many such events. GO enrichment analysis showed that the *rnp-6*-dependent DEGs suppressed by *rbm-39(S294L)* were significantly enriched in the stress response category (Extended Data Fig. 3i), indicating that this process might be associated with longevity. These results confirm that *rbm-39(S294L)* ameliorates *rnp-6(G281D)* splicing changes.

**Extended Data Fig. 4:**
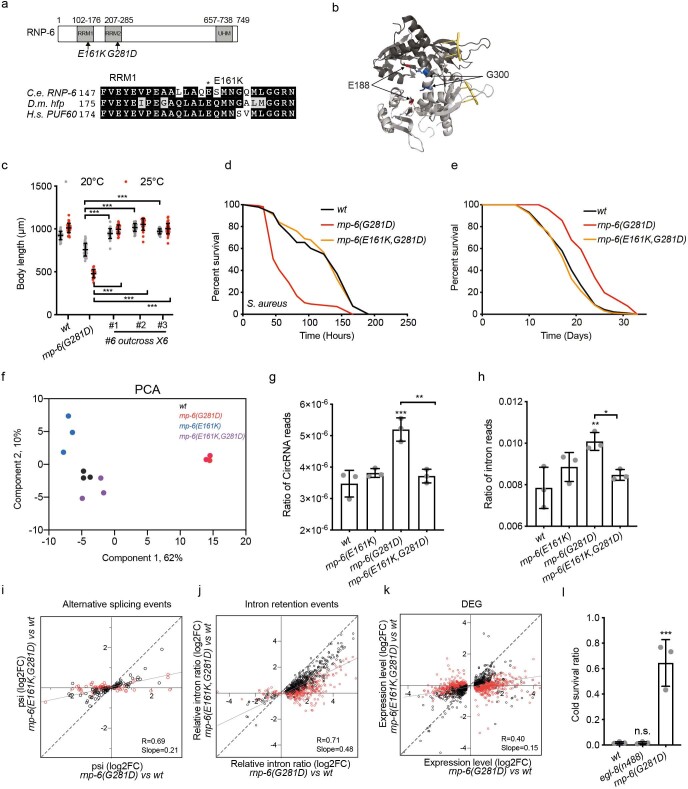
Effects of *rnp-6(E161K)* on *rnp-6(G281D)* function. a-b, Domain and protein structure of RNP-6/PUF60. Arrows in (a) indicate the mutations mentioned in this paper. The blue and red labelled amino acids are corresponding to G300 (worm G281) and E188 (worm E161), respectively. c, Effect of outcrossed *rnp-6(dh1187)* on normal development and *ts* phenotype of *rnp-6(G281D)*. n = 3. Mean ± SD. ***P < 0.001. one-way ANOVA with Dunnett adjustment. d, Representative survival plot of *rnp-6(dh1187)* upon *S. aureus* infection. n = 3. e, Representative survival plot of *rnp-6(dh1187)*. n = 3. f, PCA of RNAseq dataset for *rnp-6(E161K)*. g, Effect of *rnp-6(E161K)* on circular RNA formation. n = 3. Mean ± SEM. **P = 0.0016; ***P < 0.001. one-way ANOVA with Dunnett adjustment. h, Effect of *rnp-6(E161K)* on intron retention. n = 3. Mean ± SEM. *P < 0.0414; **P = 0.0080. one-way ANOVA with Dunnett adjustment. i-k, Effect of *rnp-6(E161K)* on alternative splicing (i), intron retention (j) and gene expression changes (k). Each dot represents the log2-transformed fold changes of an event relative to *wild-type* control. Red circles indicate the events which are significantly suppressed by *rnp-6(E161K)*. A total of 219 alternative splicing events, 826 intron retention events, and 1973 DEG events were used to generate the heat maps shown in panel i, j and k, respectively. The source data related to RNA-seq analysis are shown in Supplementary Table S10, S11 and S13. l, Effect of *egl-8(n488)* on cold tolerance. n = 3. Mean ± SD. ***P < 0.001. one-way ANOVA with Dunnett adjustment. In panels c-e and l, data from a representative experiment are shown. Source data

In addition, we found that the *rnp-6(E161K)* intragenic mutation was also a potent suppressor of *rnp-6(G281D)*, which fully restored all measured phenotypes to wild-type levels (Extended Data Fig. 4c–e). It also significantly suppressed mRNA processing as well as transcriptional changes (Extended Data Fig. 4f–k and Supplementary Tables 10–13), confirming that, like *rbm-39* mutation, restoration of splicing correlates with reversal of phenotype.

### *Egl-8* intron retention contributes to *rnp-6(G281D)* longevity

**Fig. 3:**
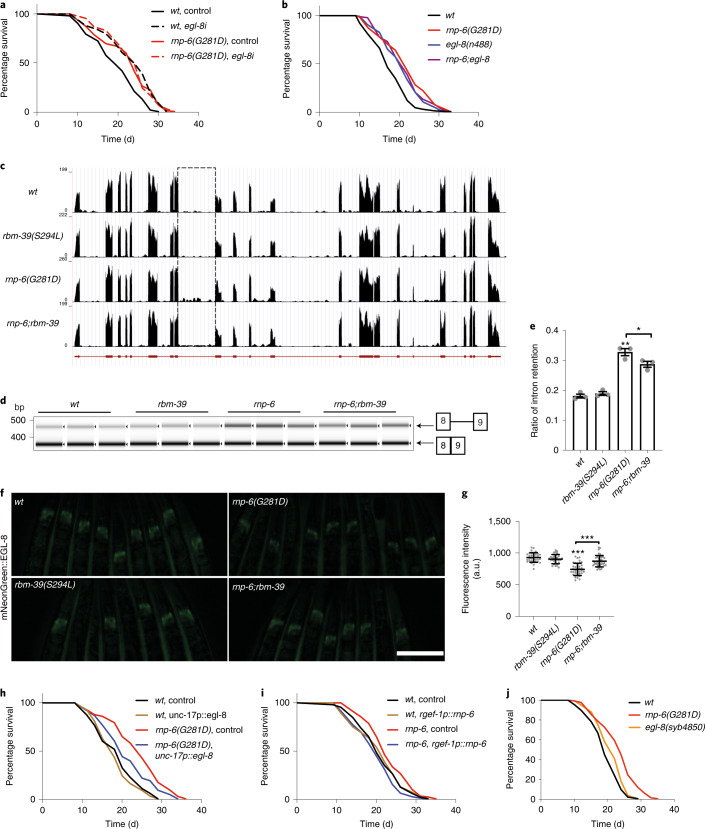
*Egl-8* intron retention promotes *rnp-6(G281D*) longevity. **a**, Representative survival plot showing the effect of *egl-8* RNAi on *rnp-6(G281D)* lifespan (*n* = 3). **b**, Representative survival plot showing the effect of *egl-8* null mutation on *rnp-6(G281D)* lifespan (*n* = 3). **c**, Representative genome browser view of *egl-8*. The dashed square indicates intron 8. **d**,**e**, Effect of *rbm-39(S294L)* on *egl-8* intron 8 retention with RT–PCR analysis (*n* = 3). Mean ± s.e.m. ^***^P < 0.001, ^*^P = 0.0307, using one-way ANOVA. **f**,**g**, Representative image and quantification of fluorescent intensity showing the effect of *rnp-6(G281D)* and *rbm-39(S294L)* on mNeonGreen::EGL-8 expression (*n* = 3). Mean ± s.d. ^***^P < 0.001 using one-way ANOVA. Scale bar, 200 μm. a.u., arbitrary units. **h**, Representative survival plot showing the effect of neuronal *egl-8* cDNA expression on *rnp-6(G281D)* lifespan (*n* = 3). **i**, Representative survival plot showing the effect of neuronally expressed *rnp-6* on *rnp-6(G281D)* lifespan (*n* = 3). **j**, Representative survival plot of *egl-8* 3′-splicing site-edited worms (*n* = 3). Detailed information of the nucleotide changes can be found in Extended Data Fig. 7a. Source data

**Extended Data Fig. 5:**
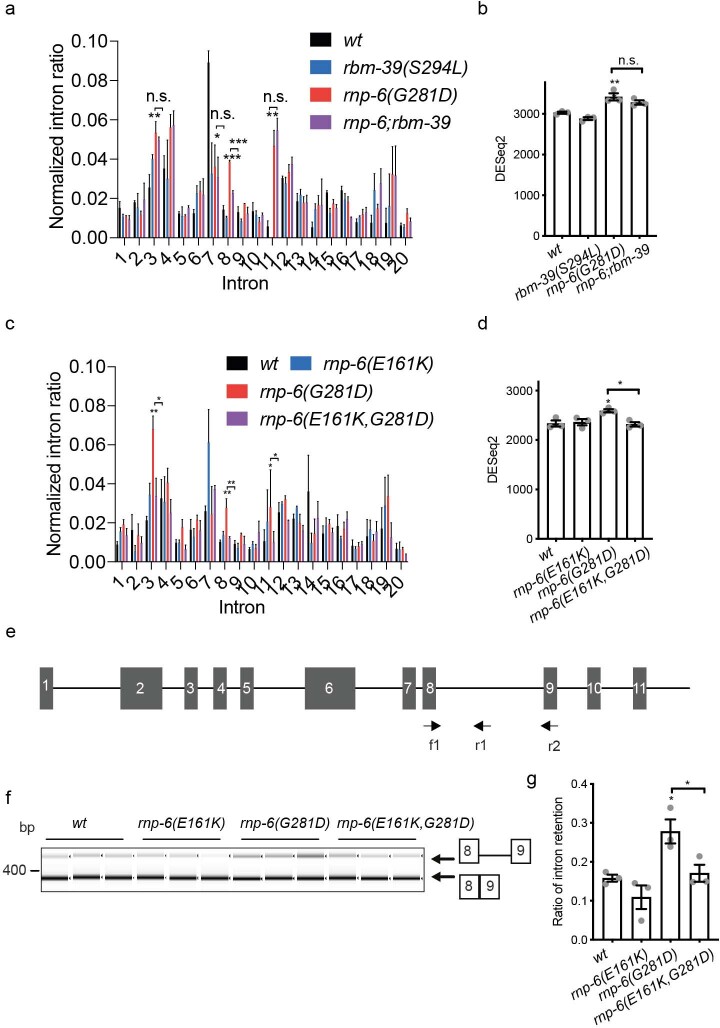
*rnp-6(G281D)* increases *egl-8* intron retention. a, Effect of *rbm-39(S294L)* on *egl-8* intron retention by RNA-seq analysis. n = 3. Mean ± SEM. ***P < 0.001; **P < 0.01; *P < 0.05. One-way ANOVA. b, Effect of *rbm-39(S294L)* on *egl-8* gene expression by RNA-seq analysis. n = 3. Mean ± SEM. **P = 0.0031;n.s. p = 0.2522. One-way ANOVA with Dunnett adjustment. c, Effect of *rnp-6(E161K)* on *egl-8* intron retention by RNA-seq analysis. n = 3. Mean ± SEM. **P < 0.01; *P < 0.05. One-way ANOVA. d, Effect of *rnp-6(E161K)* on *egl-8* gene expression by RNA-seq analysis. n = 3. Mean ± SEM. *P < 0.05. One-way ANOVA with Dunnett adjustment. e, Location of primers used in RT-PCR experiments to detect transcripts from all tissues. f-g, RT-PCR analysis of intron 8 retention in *rnp-6* intragenic mutation. n = 3. *P < 0.05, One-way ANOVA with Dunnett adjustment. Source data

**Extended Data Fig. 6:**
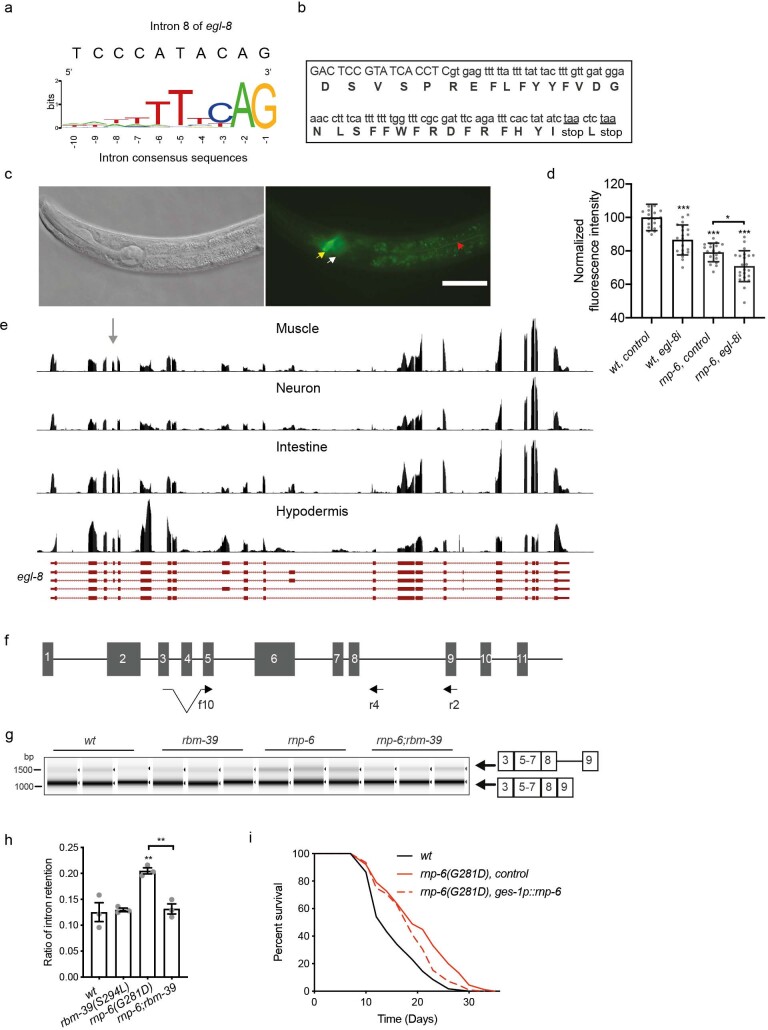
*rnp-6(G281D)* longevity is mediated partially by *egl-8* intron retention. a, Comparison of the *C. elegans* intron 3’ splice site consensus sequence with that of intron 8 of *egl-8*. Positions are numbered with respect to the splice sites. b, Effect of intron 8 retention. Uppercase indicates exon 8 and lower case denotes intron 8. c, Expression pattern of *egl-8*. Yellow arrow and white arrow indicate nerve ring and head neurons, respectively. Red arrow head indicates intestinal adherens junctions. scale bar, 50 μm. d, Effect of *egl-8* RNAi knock down on *egl-8* expression in neurons. n = 3. Mean ± SD. *P = 0.0111, ***P < 0.001. One-way ANOVA with Tukey adjustment. e, RNA-Seq coverage tracks for *egl-8* in different tissues. The exon 4 (grey arrow) is mainly skipped in neurons. Four repeats are shown for each tissue. Raw data source: PRJNA400796. f, Primers used for RT-PCR experiments to detect transcripts from neurons. g-h, RT-PCR analysis of intron retention of neuronal isoforms. n = 3. Mean ± SEM. **P < 0.01. One-way ANOVA with Dunnett adjustment. i, Representative survival plot of *rnp-6* intestine-specific rescue transgenic worms. n = 3. In panels c-d and i, data from a representative experiment are shown. Source data

To decipher the downstream mechanisms by which the RNP-6/RBM-39 complex regulates longevity, we focused on splicing events. In particular, intron retention is an important but not well-understood mechanism of gene expression regulation^23^. It is mostly associated with downregulation of gene expression via nonsense-mediated decay^24^ and has recently emerged as an important splicing feature in both normal aging and longevity interventions^4,25,26^. To reveal functionally relevant targets for the RNP-6/RBM-39 complex, we focused on intron retention induced by *rnp-6(G281D)* and restored by *rbm-39(S294L)*. We narrowed down the list of candidates to 44 events by cross-referencing with the *rnp-6(E161K)* revertant and manual curation in the genome browser (Supplementary Table 14). These 44 events correspond to 42 genes and, notably, all showed increased intron retention in *rnp-6(G281D)*. We performed RNAi knockdown to assess the impact on wild-type lifespan, reasoning that both RNAi and intron retention should result in partial loss of function. Of those genes tested, we found one candidate, *egl-8*, with a knockdown that yielded significant lifespan extension in wild-type, but did not further extend *rnp-6(G281D)* longevity (Fig. 3a). We further confirmed this genetic interaction with an *egl-8(n488)* null allele (Fig. 3b). However, the *egl-8* null did not recapitulate *rnp-6(G281D)* cold tolerance (Extended Data Fig. 4l), suggesting separatable mechanisms for cold tolerance and longevity. *Egl-8* encodes an ortholog of human PLCB4. It plays vital physiological roles in neurotransmission^27,28^, lifespan and infection response in *C. elegans*^29,30^, although the underlying molecular mechanisms are not well understood. RNA-seq data showed that *rnp-6(G281D)* altered the retention of several introns within *egl-8* (Fig. 3c and Extended Data Fig. 5a) without decreasing the total *egl-8* mRNA level (Extended Data Fig. 5b). Importantly, both *rbm-39(S294L)* and *rnp-6(E161K)* suppressed intron 8 retention (Extended Data Fig. 5a,c). Reverse transcription (RT)–PCR experiments also validated these results (Fig. 3d,e and Extended Data Fig. 5e–g). Intron 8 harbors a weak noncanonical splice acceptor site (Extended Data Fig. 6a) and its retention introduces a premature stop codon in the transcript (Extended Data Fig. 6b), which could either result in mRNA degradation by nonsense-mediated mRNA decay or give rise to a nonfunctional truncated protein. To examine its expression, we tagged endogenous EGL-8 with mNeonGreen at the N terminus. The mNeonGreen-tagged EGL-8 was mainly detected in head neurons as well as intestinal adherens junctions (Extended Data Fig. 6c), in agreement with previous immunofluorescence staining results^27^. As expected, the expression levels of EGL-8 in neurons and the nerve ring were significantly lower in *rnp-6(G281D)* compared with that in wild-type controls, and *rbm-39(S294L)* efficiently restored EGL-8 expression levels back to wild-type (Fig. 3f,g), consistent with a restoration of intron removal. Notably, neural EGL-8 expression levels were similarly reduced by approximately 20% in both *rnp-6(G281D)* and *egl-8i* (Extended Data Fig. 6d), in line with their similar impact on longevity. Furthermore, neuronal expression of *rnp-6*^*+*^ or the fully spliced *egl-8*^*+*^ complementary DNA suppressed *rnp-6(G281D)* longevity (Fig. 3h,i). To examine *egl-8* intron retention in neurons, we designed primers to detect neuronal *egl-8* transcripts based on published tissue-specific RNA-seq data^31^ (Extended Data Fig. 6e,f). RT–PCR results showed that *rnp-6(G281D)* caused more pronounced changes in neuronal *egl-8* intron retention and *rbm-39(S294L)* completely restored the defects (Extended Data Fig. 6g,h). These findings are consistent with the idea that *rnp-6(G281D)* promotes longevity via intron retention of *egl-8* within the nervous system. In addition, intestinal expression of *rnp-6* also partially rescued the *rnp-6(G281D)* lifespan (Extended Data Fig. 6i), indicating that the gut also contributes to *rnp-6*-mediated longevity.

**Extended Data Fig. 7:**
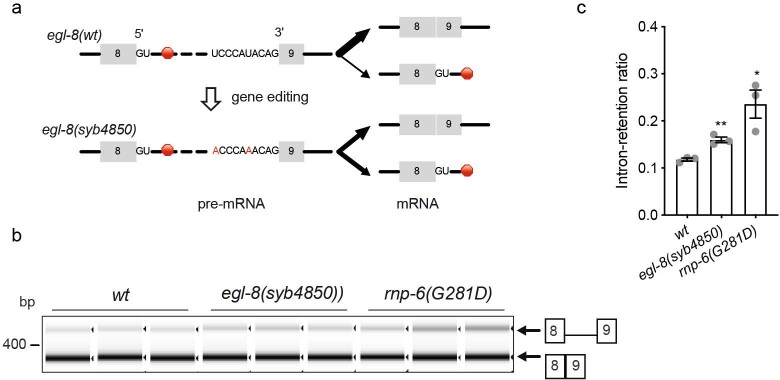
*egl-8* intron retention is sufficient for lifespan extension. a, Schematic workflow of gene editing design for *egl-8* 3’splicing sites. b-c, RT-PCR analysis of intron retention in *egl-8* gene editing mutant. n = 3. Mean ± SEM. **P = 0.0038; *P = 0.017. Two-tailed unpaired t test. Source data

To further test whether *egl-8* intron retention is sufficient for lifespan extension, we generated two T–A *cis*-acting mutations that weakened the intron 8 3′-splice acceptor site, using the intron consensus sequence as reference (Extended Data Figs. 6a and 7a). Although these nucleotide substitutions were not as potent as *rnp-6(G281D)* in disrupting splicing, they nevertheless caused a modest but significant increase in intron 8 retention (Extended Data Fig. 7b,c) and extended lifespan (Fig. 3j). Taken together, these data indicate that *egl-8* intron retention contributes to lifespan extension.

### *Rnp-6(G281D)* inhibits mTORC1 signaling through decreased EGL-8 function

**Fig. 4:**
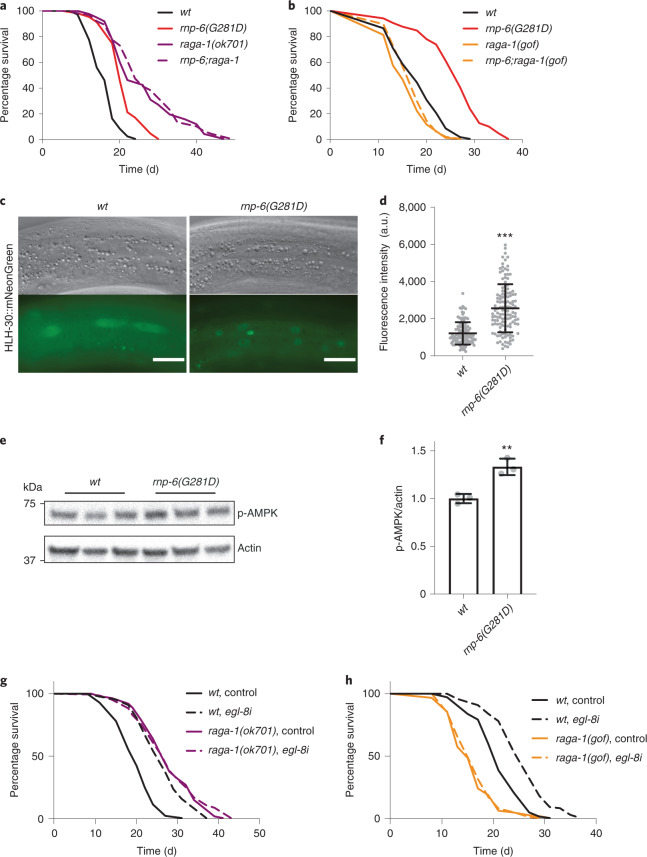
*Rnp-6(G281D*) inhibits mTORC1 signaling. **a**, Representative survival plot of *raga-1* loss of function *(ok701)* on *rnp-6(G281D)* lifespan (*n* = 3). **b**, Representative survival plot of *raga-1* gain of function *(gof)* on *rnp-6(G281D)* lifespan (*n* = 3). **c**,**d**, *Rnp-6(G281D)* alters HLH-30/TFEB nuclear localization (*n* = 3). Mean ± s.d. ^***^P < 0.001 using two-tailed, unpaired Student’s *t*-test. Scale bars in **c**, 20 μm. **e**,**f**, *Rnp-6(G281D)* alters AAK-2/AMPK phosphorylation (*n* = 3). Mean ± s.e.m. ^**^P = 0.0044, using two-tailed, unpaired Student’s *t*-test. **g**, Representative survival plot showing the effect of *egl-8 RNAi* on lifespan of *raga-1* loss-of-function mutants (*n* = 3). **h**, Representative survival plot showing the effect of *egl-8 RNAi* on lifespan of *raga-1* gain-of-function mutants (*n* = 3). Source data

**Extended Data Fig. 8:**
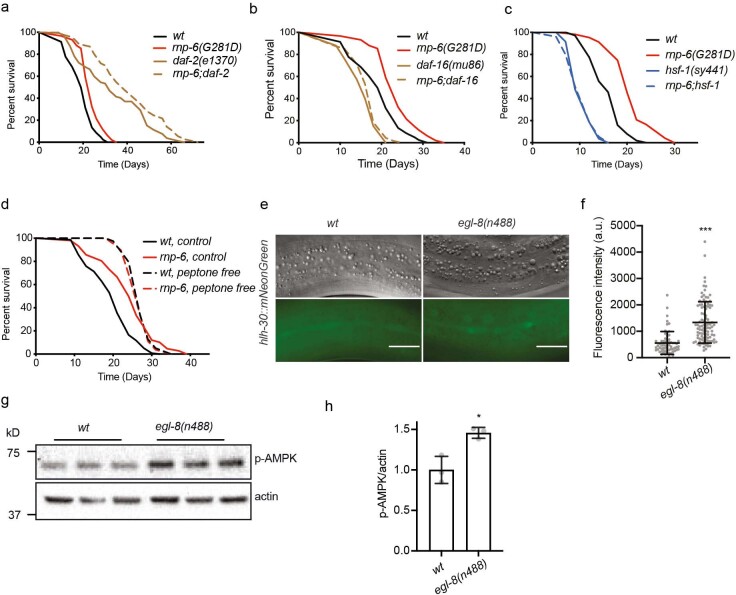
*rnp-6(G281D)* inhibits mTORC1 signaling. a, Effect of *daf-2* reduction-of-function mutation on *rnp-6(G281D)* lifespan. n = 2. b-c, Effect of *daf-16* and *hsf-1* loss-of-function mutation on *rnp-6(G281D)* lifespan. n = 3. d, Effect of peptone deprivation on lifespan. n = 2. e-f, Effect of *egl-8(n488)* on HLH-30 nuclear localization. n = 3. Mean ± SD. ***P < 0.001. Two-tailed unpaired t-test. g-h, Effect of *egl-8(n488)* on AMPK phosphorylation. n = 3. Mean ± SEM. *P = 0.0118. Two-tailed unpaired t-test. In panels a-f, data from a representative experiment are shown. Source data

To identify potential signaling pathways in which *rnp-6* might act, we performed genetic epistasis analysis, first focusing on two major conserved longevity pathways: reduced insulin/insulin growth factor (IGF) (*daf-2*, insulin/IGF receptor) and mTORC1 inhibition (*raga-1*, core component of the lysosomal amino acid-sensing machinery^32^). We found that *raga-1* but not *daf-2* longevity was nonadditive with *rnp-6* (Fig. 4a and Extended Data Fig. 8a), suggesting that *rnp-6*(*G281D)* might work in the same pathway as mTORC1. In accord with this view, the *raga-1* gain-of-function transgene, which shortens the wild-type worm lifespan^33^, completely abolished *rnp-6(G281D)* longevity (Fig. 4b), suggesting that *rnp-6* acts upstream of *raga-1* to promote mTORC1 signaling activity. Loss-of-function mutations in transcription factors FOXO/DAF-16 and HSF1/HSF-1, which mediate the output of reduced mTORC1 longevity^34,35^, also completely abrogated *rnp-6(G281D)* longevity (Extended Data Fig. 8b,c). Furthermore, *rnp-6(G281D)* longevity was also nonadditive with dietary restriction (Extended Data Fig. 8d), another longevity regimen inhibiting mTORC1 (ref. ^36^). To obtain further evidence, we tested whether molecular outputs of mTORC1 signaling were also altered in the *rnp-6(G281D)* mutant. Downregulation of mTORC1 signaling results in enhanced nuclear accumulation of HLH-30 (helix–loop–helix protein 30)/TFEB (transcription factor EB)^37,38^ and increased levels of phosphorylated AAK-2/AMPK (AMP-activated protein kinase)^39^. Consistently, we observed a significant increase in both HLH-30 nuclear localization (Fig. 4c,d) and AAK-2 phosphorylation (Fig. 4e,f) in *rnp-6(G281D)* mutants. Altogether, our results indicate that *rnp-6(G281D)* inhibits mTORC1 signaling activity through *raga-1*.

As EGL-8 serves as a downstream target of *rnp-6*, we wondered whether it also interacts with the mTORC1 signaling pathway in regulating longevity. Similar to *rnp-6(G281D)*, *egl-8(n488)* loss-of-function mutation significantly inhibited mTORC1 activity as indicated by increased HLH-30 nuclear localization (Extended Data Fig. 8e,f) and AMPK phosphorylation (Extended Data Fig. 8g,h). Furthermore, *egl-8i* did not further extend the lifespan of *raga-1* null mutants (Fig. 4g), whereas *raga-1* gain-of-function transgene completely suppressed *egl-8i-*induced longevity (Fig. 4h). These results demonstrate that *egl-8* acts upstream of *raga-1*, linking *rnp-6* to mTORC1 signaling, and are consistent with previous studies showing that phospholipases can control mTORC1 activity via the generation of phosphatidic acid^40^.

### PUF60 regulates mammalian mTORC1 signaling

**Fig. 5:**
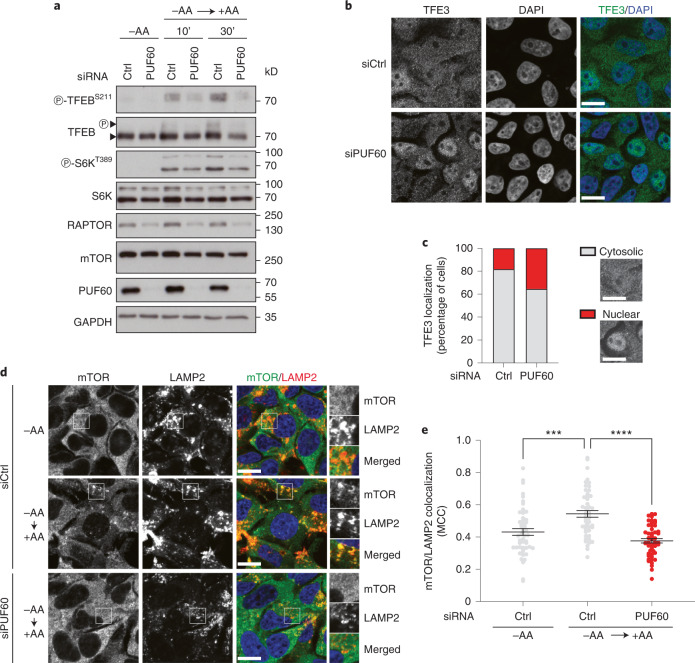
PUF60 regulates mTORC1 signaling in mammalian cells. **a**, Effect of PUF60 knockdown on mTORC1 activity. Western blots with lysates from HEK293FT cells that are transiently transfected with siRNAs targeting PUF60 or a control RNAi duplex (Ctrl), and treated with medium containing or lacking amino acids (AA), in starvation (–AA) or add-back (±AA) conditions, probed with the indicated antibodies. Arrowheads indicate bands corresponding to different TFEB forms (*n* = 3 independent experiments). P, phosphorylated form. **b**, PUF60 knockdown enhances nuclear TFE3 localization. TFE3 localization analysis in HEK293FT, transiently transfected with siRNAs targeting PUF60 or a control RNAi duplex (Ctrl), using confocal microscopy. Nuclei stained with DAPI (*n* = 3 independent experiments). Scale bars, 10 μm. **c**, Scoring of TFE3 localization from **b**. Individual cells were scored for nuclear or cytosolic TFE3 localization, as indicated in the example images. A representative experiment of three independent replicates is shown. **d**,**e**, PUF60 knockdown reduces lysosomal localization of mTORC1. Colocalization analysis is shown of mTOR with LAMP2 (lysosomal marker) in HEK293FT cells transiently transfected with siRNAs targeting PUF60 or a control RNAi duplex (Ctrl), using confocal microscopy. Magnified insets are shown to the right (*n* = 3 independent experiments (**d**)). Scale bars, 10 μm. Quantification of mTOR/LAMP2 colocalization from *n* = 50 individual cells per condition from a representative experiment of 3 independent replicates is shown in **e**. Mean ± s.d. ^***^P < 0.001, ^****^P < 0.0001, using one-way ANOVA. Source data

**Extended Data Fig. 9:**
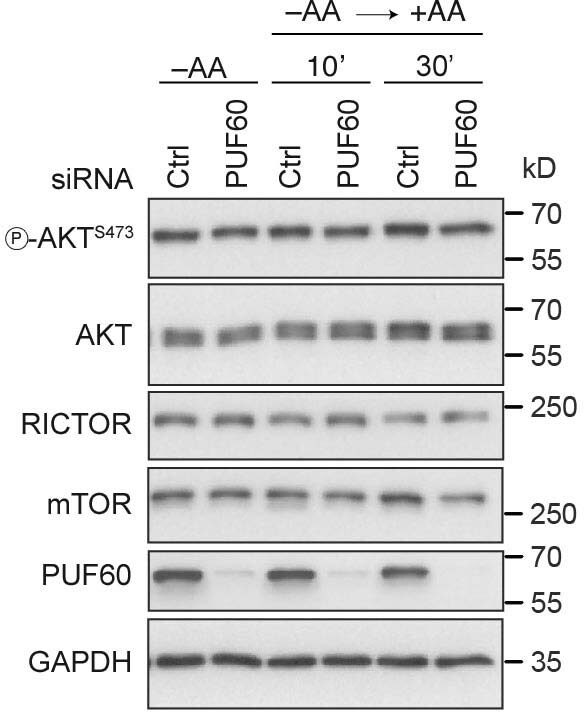
PUF60 knockdown does not influence mTORC2 activity. Immunoblots with lysates from HEK293FT WT cells, transiently transfected with siRNAs targeting PUF60 or a control RNAi duplex (Ctrl), and treated with media containing or lacking AA, in starvation (–AA) or add-back (–AA/ + AA) conditions, probed with the indicated antibodies. n = 3 independent experiments. Western blots from a representative experiment are shown. Source data

Last, we sought to understand whether the functional interaction of RNP-6 and mTORC1/RAGA-1 was evolutionarily conserved. To this end, we knocked down PUF60 by small interfering (si)RNA in HEK293FT cells, which show several characteristics that are normally observed in immature neurons^41^ and have been extensively used in studies on the mechanisms of amino acid sensing by mTORC1 (ref. ^32^). As *rnp-6* regulates mTORC1 upstream of *raga-1* (Fig. 4g,h), we surmised that PUF60 might affect amino acid signaling to mTORC1. In accord with this view, PUF60 knockdown decreased mTORC1 reactivation on amino acid re-supplementation, assayed by the phosphorylation of its direct substrates S6K (ribosomal protein S6 kinase β1) and TFEB (Fig. 5a), without influencing mTORC2 activity (assayed by Akt phosphorylation) (Extended Data Fig. 9). Accordingly, we consistently observed decreased RAPTOR (regulatory-associated protein of mTOR) protein levels on PUF60 knockdown, whereas the levels of the respective mTORC2 core component, RICTOR, or of mTOR itself, were largely unaffected (Fig. 5a and Extended Data Fig. 9). In line with the *C. elegans* results, and further supporting decreased mTORC1 activity, PUF60 knockdown enhanced the nuclear localization of transcription factor E3 (TFE3) (Fig. 5b,c). As amino acid sufficiency controls mTORC1 localization to lysosomes via promoting RAPTOR binding to the lysosomal Rag GTPase dimers, we then hypothesized that PUF60 may regulate mTORC1 activity by influencing its subcellular localization. Indeed, knocking down PUF60 caused a significant drop in the colocalization of mTOR with the lysosomal marker LAMP2 (lysosome-associated membrane glycoprotein 2) in cells re-supplemented with amino acids (Fig. 5d,e). These findings reveal that PUF60 acts as a specific and integral part of the mTORC1 signaling pathway, influencing the amino acid-induced activation of mTORC1 at the lysosomal surface. Whether the mechanistic details of mTORC1 regulation by PUF60 in mammalian cells are the same as those in worms remains to be seen in future studies.

## Discussion

Messenger RNA splicing is a fundamental cellular process which has recently emerged as important to organismal aging. Although specific splicing factors and splicing events have been shown to be associated with the aging process^42,43^, the underlying molecular mechanisms remain largely unknown. Our studies provide direct evidence that a genetic mutation within a core spliceosome component promotes longevity partially through intron retention in *C. elegans*, highlighting such events in the fidelity of information processing and stress response.

*Rnp-6* encodes an essential splicing factor and the null mutation causes lethality in *C. elegans*. However, *rnp-6(G281D)* shows no overt defects under standard growth conditions at 20 °C. Our RNAi, rescue and RNA-seq experiments indicate that *rnp-6(G281D)* is a unique change or selective loss-of-function allele, which is similar to, yet distinct from, canonical functional depletion by knockdown. Notably, *rnp-6(G281D)* mutants are long-lived. In comparison, *rnp-6i* during development and from D1 of adulthood onward impair essential functions and shorten lifespan, whereas *rnp-6i* from D4 of adulthood promotes longevity. In contrast to *rnp-6* RNAi, mTOR RNAi extends life from D1 onward^44^. These findings imply that the activities of splicing factors are fine-tuned, and suggest an antagonistic pleiotropic trade-off that is beneficial early on but detrimental later in life, similar to other essential genes of autophagy^14,15^ and de-ubiquitination^45^ in *C. elegans*. Whether *rnp-6(G281D)* and D4 on *rnp-6i* work by the same precise mechanism is currently unknown.

Splicing factors form extraordinarily large and highly dynamic macromolecular assemblies to catalyze splicing. Similar to transcription factors, there are both positive and inhibitory regulators of this process^1^. From our study, we identified a lesion in splicing factor RBM-39(Ser294Leu), which forms nuclear speckles, alleviates RNP-6(Gly281Asp) defects and reverses longevity phenotypes, giving us mechanistic insight into critical targets of splicing. It is interesting that in mammalian cells RBM39, PUF60 and the large subunit of the U2AF complex, U2AF65, share a similar domain architecture and work in close proximity to regulate 3′-splice site assembly. Notably, all three proteins interact with the U2 snRNP subunit, SF3b155, through their UHM domains, and U2AF65 and RBM39 have been shown to do so cooperatively^46^. Conceivably, RBM-39(Ser294Leu) ameliorates RNP-6(Gly281Asp) by altering interactions with other splicing factors within nuclear speckles. Whether RBM39 and PUF60 bind to SF3b155 in a cooperative or competitive manner remains to be seen, but their close proximity suggests possible modes of functional interaction and highlights the importance of this specific complex in stress signaling and lifespan control.

Intron retention is a major mechanism of gene expression regulation^23^ and has recently been shown as a common feature in aging-related splicing changes^25^. Intron retention is also associated with longevity interventions, such as diet restriction, in both *C. elegans* and mice^4^. Several lines of functional evidence provide direct causal evidence that *egl-8* intron retention in neurons contributes to organismal longevity. First, *rnp-6(G281D)* mutation causes *egl-8* intron retention, reduced neural EGL-8 protein levels and longevity, in a manner restored by *rbm-39(S294L)*. Furthermore, *rnp-6(G281D)* induces a level of *egl-8* downregulation comparable to that of *egl-8* RNAi, which similarly extends life. Second, *cis*-acting mutations that weaken the *egl-8* intron 8 3′-splice site are sufficient to promote both intron retention and longevity, despite a smaller magnitude effect to *rnp-6(G281D)* itself. Third, neuronal expression of full-length *egl-8* cDNA is sufficient to suppress *rnp-6(G281D)* longevity, although gut-specific *egl-8* expression reportedly has little impact on lifespan^29,30^. By contrast, expression of *rnp-6* in gut can rescue lifespan (Extended Data Fig. 6i), suggesting that *egl-8*-independent mechanisms contribute to *rnp-6(G281D)* longevity as well. In sum, we conclude that *rnp-6(G281D)* longevity arises at least in part from *egl-8* intron retention, although other activities probably contribute.

We would like to point out that the observed ratio of intron 8 retention as measured by RNA-seq was relatively small, ranging from 1.5% in wild-type to 4% in *rnp-6(G281D)* (Extended Data Fig. 5a), raising some question about its significance. However, because of technical limitations, we obtained RNA from whole worms and thus the relevant isoforms in functionally important tissues are underestimated. Notably, *egl-8* mRNA has a highly complex, alternative splicing pattern and is expressed in multiple tissues. Despite this, the *egl-8* cDNA containing all exons when expressed in neurons can regulate lifespan throughout the body (Fig. 5h and previous reports^29,30^). In contrast to RNA-seq, the RT–PCR experiments showed greater levels of intron retention (Fig. 3e and Extended Data Fig. 6h). This apparent enrichment reflects the use of primers annealing to exon 9, which is present in the full-length cDNA used to rescue lifespan, but not in all isoforms (Extended Data Fig. 6e). In addition, it is possible that processes downstream of intron retention, such as reduced translation, may also impact expression^47,48^. Notably, it is not uncommon for relatively small changes in gene expression to influence phenotype in other settings^49,50^.

**Extended Data Fig. 10:**
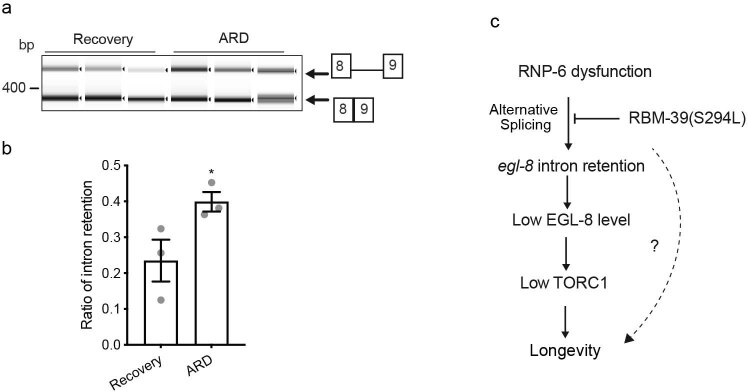
Model for *rnp-6* mediated lifespan regulation. a-b, RT-PCR analysis of *egl-8* intron retention in ARD and ARD recovery worms. n = 3. Mean ± SEM. *P = 0.03. One-tailed unpaired t-test. c, Proposed model for RNP-6/RBM-39/EGL-8/TORC1 axis in regulating longevity *in C. elegans*. It should be noted that *rnp-6/rbm-39* longevity might also be mediated by *egl-8* independent mechanisms.

Our study reveals that RNP-6 and PUF60 share an evolutionarily conserved role in regulating mTORC1 signaling, but it is not yet clear if this regulation also happens via PLCB4 in mammalian cells. This will require additional follow-up studies and the outcome could depend on cell type or signaling context. The question arises as to whether RNP-6/PUF60 regulates intron retention in a normal physiological context of cellular signaling. In accord with this idea, we also found that *egl-8* intron retention is induced under conditions of food deprivation in wild-type animals within the adult reproductive diapause^51,52^, which is reversed by re-feeding (Extended Data Fig. 10a,b), resembling the amino acid starvation/re-supplementation protocols used in cell culture assays to study the reactivation of mTORC1. Conceivably, external environmental cues or internal physiological signals may trigger intron retention events to modulate the host response. An interesting question is whether the impact of *rnp-6/rbm-39* on *egl-8* and mTORC1 signaling represents a specific signaling pathway or a broader stress response in which *egl-8* serves as a sentinel for aberrant splicing.

In summary, our results suggest a model whereby the RNP-6/RBM-39 spliceosomal complex impacts splicing fidelity to regulate mTOR signaling and longevity (Extended Data Fig. 10c). Our study implicating components of the splicing machinery working upstream of mTOR signaling may provide new approaches to manipulate this pathway in aging, metabolism and disease. Precise targeting of PUF60, and perhaps RBM39, could be used to downregulate mTORC1 signaling to confer health benefits similar to rapamycin and other rapalogs^53,54^. Conversely, as many spliceosomopathies that reduce spliceosomal function trigger growth defects^8,55–58^, it may be possible to treat these diseases with mTOR modulators.

## Methods

### *C. elegans* strains and maintenance

The following strains were used in the present study: N2 (wild-type), *rnp-6(dh1127)*, *rbm-39(syb1074)*, *rnp-6(dh1127);rbm-39(syb1074)*, *rbm-39(gk454899)*, *rnp-6(dh1127);rbm-39(gk454899)*, *egl-8(syb3661)*, *rnp-6(dh1127);egl-8(syb3661)*, *egl-8(syb4850)*, *raga-1(ok701),* *rnp-6(dh1127);raga-1(ok701)*, *egIs12[raga-1(gf);Pofm-1::GFP], (rnp-6(dh1127), egIs12[raga-1(gf);Pofm-1::GFP])*, *daf-2(e1370)*, *rnp-6(dh1127);daf-2(e1370)*, *daf-16(mu86)*, *rnp-6(dh1127);daf-16(mu86)*, *(wt, dhEx1132[rnp-6p::rnp-6, myo-2p::GFP])*, *(rnp-6(dh1127), dhEx1132[rnp-6p::rnp-6, myo-2p::GFP])*, *(wt, dhEx1139 dhEx1139[rnp-6p::FLAG::HA::GFP::rnp-6b cDNA::unc-54 3*′*-UTR, myo-3::mcherry])*, *(rnp-6(dh1127)*, *dhEx1139[rnp-6p::FLAG::HA::GFP::rnp-6b cDNA::unc-54 3*′*-UTR, myo-3::mcherry])*, *(wt, dhEx1147[rnp-6p::FLAG::HA::GFP::rnp-6b(G281D) cDNA::unc-54 3*′*-UTR, myo-3::mCherry], (rnp-6(dh1127))*, *dhEx1147[rnp-6p::FLAG::HA::GFP::rnp-6b(G281D) cDNA::unc-54 3*′*-UTR, myo-3::mCherry]*, *(wt, dhEx1159[ges-1p::gfp::rnp-6b cDNA(wt), myo-3p::mCherry])*, *(rnp-6(dh1127)*, *dhEx1159[ges-1p::gfp::rnp-6b cDNA(wt), myo-3p::mCherry]), (wt, dhEx1208[unc-17p::gfp::egl-8, myo-3p::mCherry])*, *(rnp-6(dh1127)* and *dhEx1208[unc-17p::gfp::egl-8, myo-3p::mCherry])*. Detailed information for strains used in the present study can be found in Supplementary Table 18. All mutant strains obtained from the Caenorhabditis Genetics Center (CGC) or National BioResource Project (NBRP) were outcrossed with our N2 at least twice before experiments. Worms were maintained at 20 °C following standard procedures^59^. For all experiments, synchronization of the animals was done through the egg laying.

### Cell culture and treatments

All cell lines were grown at 37 °C, 5% CO_2_. Human female embryonic kidney HEK293FT (Invitrogen, catalog no. R70007; RRID: CVCL_6911) cells were cultured in high-glucose Dulbecco’s modified Eagle’s medium (DMEM; Thermo Fisher Scientific, catalog no. 41965039), containing 10% fetal bovine serum (FBS) and 1% penicillin–streptomycin. The cells were purchased from Invitrogen before the start of the project. Their identity was validated by the Multiplex human Cell Line Authentication test (Multiplexion GmbH), which uses an SNP typing approach and was performed as described at www.multiplexion.de. All cell lines were regularly tested for *Mycoplasma* contamination using a PCR-based approach and were confirmed to be *Mycoplasma* free. Amino acid (AA) starvation/re-addition experiments were performed as described previously^60^. In brief, customized starvation media were formulated according to the Gibco recipe for high-glucose DMEM, specifically omitting the amino acids. The media were filtered through a 0.22-μm filter device and tested for proper pH and osmolality before use. For the respective AA-replete treatment medium, commercially available high-glucose DMEM was used (Thermo Fisher Scientific, catalog no. 41965039). All treatment media were supplemented with 10% dialyzed FBS. For this purpose, FBS was dialyzed against 1× PBS through 3,500 molecular mass cutoff dialysis tubing. For AA starvation (–AA), culture media were replaced with starvation media for 1 h. For AA add-back experiments (+AA), cells were first starved as described above and then starvation media were replaced with +AA treatment media for 10 or 30 min.

### Plasmid construction and transgenesis

For *rnp-6* rescue plasmid*, rnp-6* promoter (3,135 bp) was amplified from the N2 genome and inserted into a pDC4 vector-generated *rnp-6p::FLAG::HA::GFP::::unc-54* 3′-UTR construct. Then, *rnp-6b* cDNA was amplified from N2 cDNA and cloned into this plasmid to generate a *rnp-6p::FLAG::HA::GFP::rnp-6b cDNA::unc-54* 3′-UTR rescue plasmid. Site-directed mutagenesis (Q5 Site-Directed Mutagenesis Kit, New England Biolabs (NEB)) was performed to incorporate Gly281Asp point mutation to generate *rnp-6p::FLAG::HA::GFP::rnp-6b(G281D) cDNA::unc-54* 3′-UTR plasmid. To generate neuronal rescue plasmid, *rnp-6* promoter was replaced by neuronal-specific promoter *rgef-1* (2,670 bp). The *unc-17p::gfp::egl-8* plasmid is a kind gift from S. Nurrish (Harvard Medical School). The microinjection experiments were performed according to a standard protocol^61^: 10 ng μl^−1^ of plasmid of interest together with a 5-ng μl^−1^ of co-injection marker plasmid (*myo-3p::mCherry*) were injected into the gonads of young adult-stage worms. Positive offspring were singled to maintain stable lines. PCR primers related to these plasmids are available in Supplementary Table 15.

### EMS mutagenesis screen and mapping

The cold resistance-longevity screen that identified *rnp-6(G281D)* had been performed previously^13^. The heat-sensitivity suppressor screen was done on an *rnp-6(G281D)* mutant background. Briefly, ~1,000 L4 larvae worms (P0) were exposed to 0.15% ethyl methane sulfonate (EMS, Sigma-Aldrich) in M9 buffer for 4 h at room temperature. After overnight recovery, young adult P0 animals were transferred to new plates for egg laying at 20 °C. After 3 d of growing, adult F1 worms were bleached and eggs were seeded on nematode growth medium (NGM) plates and incubated at 25 °C. After 3 d, F2 worms that reached adulthood were singled and maintained at 20 °C. False-positive hits were excluded by re-testing heat sensitivity in F4/F5 generations. The *rnp-6(G281D)* animals were used as a negative control in all heat-sensitivity assays. Hawaiian SNP mapping and whole-genome sequence were used to map the causative mutation^62^. The *rnp-6(G281D)* mutation was first introduced to Hawaiian CB4856 by 6× outcrossing. Then, the EMS mutants were crossed with Hawaiian males that carry the *rnp-6(G281D)* mutation. Eggs of the F1 generation worms were grown at 25 °C and adult F2 were singled after 3 d. The heat-resistant strains were then pooled together, and genomic DNA was purified using Gentra Puregene Kit (QIAGEN). The pooled DNA was sequenced on an Illumina HiSeq platform (paired-end 150 nt). An MiModD pipeline (https://celegans.biologie.uni-freiburg.de/?page_id=917) was used to identify the mutations. The WS220/ce10 *C. elegans* assembly was used as reference genome for annotation. The causative mutations were confirmed by either CRISPR–Cas9 or multiple outcross. *Dh1183* and *dh1187* were identified as *rbm-39(S294L)* and *rnp-6(E161K)*, respectively. The causative genes for the other 11 mutants remain unclear.

### Stress tolerance assays

For cold tolerance assays, worms were synchronized and grown on standard NGM plates. When the worms reached the young adult stage, they were transferred to a 2 °C incubator for 24 h. The worms were recovered at room temperature for 4 h and the number of alive and dead worms were scored. The cold survival ratio was measured as the ratio of the number of live worms to the number of total worms. At least 60 worms from each genotype were used in the assay for each biological replicate. Three independent repeats were performed. For the heat-sensitivity assay, synchronized eggs from different genotypes were grown on standard NGM plates at 20 °C or 25 °C. After 3 d, images of the worms were captured. Body length or body area was measured by ImageJ software. At least 15 worms from each genotype were used for each biological replicate. Three independent repeats were performed except in Extended Data Fig. 2a,c in which two repeats were performed.

### Protein alignments and phylogenetic analysis

Homologs of RNP-6 and RBM-39 were identified from Wormbase (https://wormbase.org). A T-Coffee algorithm^63^ was used to align RNP-6, RBM-39 and their homologs from different species. Phylogenetic analysis of RBM-39 and its homologs was done with Phylogeny.fr^64^. Protein sequences of *Homo sapiens* RBM39 (UniProt: Q14498), *C. elegans* RBM-39 (UniProt: Q9N368) and *Drosophila melanogaster* Caper (UniProt: Q9VM49) were used in Fig. 2d. Protein sequences of *H. sapiens* PUF60 (UniProt: Q9UHX1), *C. elegans* RNP-6 (UniProt: Q9N3S4) and *D. melanogaster* Hfp (UniProt: Q8T6B9) were used in Supplementary Fig. 2b. Protein sequences of *C. brenneri* RBM-39 (UniProt: G0NLU2), *C. elegans* RBM-39 (UniProt: Q9N368), *Strongyloides ratti* SRAE (UniProt: A0A090LFF6), *C. briggsae* RBM-39 (UniProt: A8XIX5), *C. japonica* RBM-39 (UniProt: A0A2H2IF69), *C. remanei* RBM-39 (UniProt: E3MXT8), *Brugia malayi* RBM-39 (UniProt: A0A4E9ESP8), *Trichus muris* RBM39 (UniProt: A0A5S6R6A6), *Xenopus tropicalis* RBM39 (UniProt: Q566M5), *Rattus norvegicus* RBM39 (UniProt: Q5BJP4), *Pan troglodytes* RBM39 (UniProt: A0A2I3RX33), *Schizosaccharomyces pombe* Rsd1 (UniProt: O13845), *Mus musculus* RBM39 (UniProt: Q8VH51), *H. sapiens* RBM39 (UniProt: Q14498), *Gallus gallus* RBM-39 (UniProt: E1BRU3), *D. melanogaster* Caper (UniProt: Q9VM49), *Bos taurus* RBM39 (UniProt: A0A3Q1LWZ4), *Anopheles* *gambiae* RBM39 (UniProt: Q7PN29), *Danio rerio* RBM39 (UniProt: Q58ER0), *Canis lupus* RBM39 (UniProt: E2R4L0) and *Pristionchus pacificus* RBM39 (UniProt: H3FJ10) were used in Extended Data Fig. 2d,e. The crystal structure of PUF60 in Extended Data Fig. 4b was adapted from the literature^16^.

### CRISPR–Cas9 mutant and reporter strains

To generate hemagglutinin (HA)-tagged *rnp-6* strains, guide RNAs were selected by using the web tool (https://zlab.bio/guide-design-resources). Single guide (sg)RNAs were synthesized with EnGen sgRNA Synthesis Kit (NEB, catalog no. E3322) by following the manufacturer’s protocol. CRISPR–Cas9 insertion was generated by following a co-CRISPR strategy^65^. *Dpy-10* was used as a marker to enrich potential hits. RNP complexes containing sgRNA, Cas9 and repair templates were annealed at 37 °C for 15 min before injection. The primers used in the present study are listed in Supplementary Table 15. GFP-tagged RNP-6 strains (*rnp-6(syb645), rnp-6(syb626)*), mKate2-tagged RBM-39 strains (*rbm-39(syb1527), rbm-39(syb1545)*), mNeonGreen-tagged EGL-8 strain (*egl-8(syb3661)*), *rbm-39(S294L)* mutant strain (*rbm-39(syb1074)*) and *egl-8* intron 8 3′-splicing site-edited strain (*egl-8(syb4850)*) were generated by Sunybiotech (https://www.sunybiotech.com). All the strains were validated by Sanger sequencing.

### Lifespan

All lifespans were performed at 20 °C unless otherwise noted. Worms were allowed to grow to the young adult stage on standard NGM plates with OP50. For each genotype, ~150 young adults were transferred to NGM plates with OP50 supplemented with 10 µM FUDR. For lifespan experiments with *egl-8(n488)*, a final concentration of 50 µM FUDR was added to NGM plates. Survival was monitored every other day. Worms that did not respond to gentle touch by a worm pick were scored as dead and were removed from the plates. Animals that crawled off the plate or had ruptured vulva phenotypes were censored. All lifespan experiments were blinded and performed at least 3× independently unless otherwise noted. Graphpad Prism (9.0.0) was used to plot survival curves. Survival curves were compared and *P* values were calculated using the log(rank) (Mantel–Cox) analysis method. Complete lifespan data are available in Supplementary Table 16.

### Infection assay

*Staphylococcus aureus* (strain MW2) was grown in Tryptic Soy Broth medium at 37 °C with gentle shaking overnight. Then, 100 μl of the bacterial culture was seeded and spread all over the surface of the trypticase soy agar plate with 10 μg ml^−1^ of nalidixic acid. The plates were allowed to grow overnight at 37 °C. On the next day, the plates were left at room temperature for at least 6 h before the infection experiments. Around 25 synchronized young adult worms were transferred to the plates. Three technical replicate plates were set up for each condition. Worms were treated with 100 μM FUDR from L4 stage to prevent internal hatching during experiments. The plates were then incubated at 25 °C to initiate the infection experiment. Scoring was performed every day. Worms were scored as dead if the animals did not respond to gentle touch by a worm pick. Worms that crawled off the plates or had ruptured vulva phenotypes were censored from the analysis. All *C. elegans* killing assays were performed 3× independently unless otherwise stated. At least 60 worms per genotype were included at the start of the assay for each replicate. Genotypes were blinded for all *C. elegans* infection survival experiments to eliminate any investigator-induced bias. Results of each biological replicate of infection survival experiments can be found in Supplementary Table 17.

### RNAi in *C. elegans*

RNAi experiments were performed as previously described^13^. *E. coli* HT115 and *E. coli OP50(xu363)* bacterial strains were used in the present study. The HT115 bacteria were from the Vidal or Ahringer library. The *OP50(xu363)* competent bacteria were transformed with double-stranded RNA expression plasmids, which were extracted from the respective HT115 bacterial strains. The RNAi bacteria were grown in lysogeny broth medium supplemented with 100 µg ml^−1^ of ampicillin at 37 °C overnight with gentle shaking. The culture was spread on RNAi plates, which are NGM plates containing 100 µg ml^−1^ of ampicillin and 0.4 mM isopropyl β-d-1-thiogalactopyranoside. RNAi-expressing bacteria were allowed to grow on the plates at room temperature for 2 d. RNAi was initiated by letting the animals feed on the desired RNAi bacteria. RNAi experiments related to Fig. 1d–f and Extended Data Fig. 2g,h were done with *OP50(xu363)* bacteria. For RNAi lifespan experiments related to Fig. 3a,m,n, worms were grown on HT115 RNAi bacteria from the egg until day 1 adulthood and then transferred to NGM plates seeded with OP50.

### RNA extraction and cDNA synthesis

*C. elegans* was lysed with QIAzol Lysis Reagent. RNA was extracted using chloroform extraction. The samples were then purified using RNeasy Mini Kit (QIAGEN). Purity and concentration of the RNA samples were assessed using a NanoDrop 2000c (peqLab). The cDNA synthesis was performed using an iScript cDNA synthesis kit (BioRad). Standard manufacturers’ protocols were followed for all mentioned commercial kits.

### RNA-seq and bioinformatic analysis

Total RNA, 1 µg, was used per sample for library preparation. The protocol of Illumina Tru-Seq-stranded RiboZero was used for RNA preparation. After purification and validation (2200 TapeStation, Agilent Technologies), libraries were pooled for quantification using the KAPA Library Quantification kit (Peqlab) and the 7900HT Sequence Detection System (Applied Biosystems). The libraries were then sequenced with the Illumina HiSeq4000 sequencing system using paired-end 2× 100-bp sequencing protocol. For data analysis, the Wormbase genome (WBcel235_89) was used for alignment of the reads. This was performed with Hisat v.2.0.4 (ref. ^66^). DEGs between different samples were identified using Stringtie (v.1.3.0)^67^, followed by Cufflinks (v.2.2)^68^. The enrichment visualization was performed with WormCat 2.0 (ref. ^69^). *P* values were calculated from Fisher’s exact tests and adjusted with Bonferroni’s multiple hypothesis test. *P* < 0.05 was defined as significant. An SAJR pipeline^70^ was used for splicing analysis. Significant splicing changes were defined as those with *P* < 0.05 after adjusting for multiple testing using the Benjamini–Hochberg correction. For intron retention analysis, Bedtools coverage (v.2.29.0) was used to count intron and total gene expression. IBB (v.20.06; R v.4.0.3)^71^ was used to calculate differential intron expression. DCC/CircTest (v.0.1.0) pipeline^72^ was performed to quantify circular RNA expression. Weblogo^73^ was used to generate intron 3′-splice site consensus sequence logos (Extended Data Fig. 6a). A total of 9,484 3′-splice site sequences from SAJR analysis were used. Row *z*-score heat maps (Fig. 2k–m) were generated by using the iHeatmap function from Flaski (v.2.0.0) (10.5281/zenodo.5254193). Adjusted *P* value/*q* value <0.05 is considered to be significant for SAJR and DEG analysis; *P* < 0.001 is considered to be significant for intron retention analysis.

### Alternative splicing PCR assay

Phusion Polymerase (Thermo Fisher Scientific) was used to amplify the *egl-8, tos-1 and tcer-1* segments. PCR reactions were cycled 30× with an annealing temperature of 53 °C. RT–PCR products were either analyzed by using TapeStation 2200 (Agilent, Revision A.02.02 (SR1)) or visualized with ChemiDoc Imager (BioRad, ChemiDoc MP, Image Lab 6.1) after staining with Roti-GelStain (Carl Roth). Sequences of the primers used in the RT–PCR assays are available in Supplementary Table 15.

### Western blotting

For *C. elegans* samples, animals were first washed with M9 buffer. Worm pellets were resuspended in radioimmunoprecipitation assay (RIPA) buffer supplemented with cOmplete Protease Inhibitor (Roche) and PhosSTOP (Roche) and snap-frozen in liquid nitrogen. Thawed samples were lysed using the Bioruptor Sonication System (Diagenode). Protein samples were then heated to 95 °C for 10 min in Laemmli buffer with 0.8% 2-mercaptoethanol to denature proteins. Samples were loaded on 4–15% Mini PROTEAN TGXTM Precast Protein Gels (BioRad) and electrophoresis was performed at a constant voltage of 200 V for around 40 min. After separation, the proteins were transferred to poly(vinylidene fluoride) membranes using Trans-Blot TurboTM Transfer System (BioRad). Then, 5% bovine serum albumin (BSA) or 5% milk in Tris-buffered saline and Tween-20 (TBST) was used for blocking of the membranes. After antibody incubations (anti-HA 1:1,000; anti-phospho-AMPKα (Thr172) 1:2,000; anti-red fluorescent protein 1:2,000; anti-beta actin, 1:5,000; anti-FLAG, 1:2,000; anti-mouse horseradish peroxidase (HRP), 1:5,000; anti-rabbit HRP, 1:5,000; and anti-rat HRP, 1:5,000) and washing with TBST buffer, imaging of the membranes was performed with ChemiDoc Imager (BioRad, ChemiDoc MP, Image Lab 6.1). Western Lightning Plus Enhanced Chemiluminescence Substrate (PerkinElmer) was used as the chemiluminescence reagent. For western blotting analyses using HEK293FT samples, cells were washed once in-well with serum-free DMEM (Thermo Fisher Scientific, catalog no. 41965039) to remove FBS, and lysed in 250 µl of lysis buffer (50 mM Tris, pH 7.5, 1% Triton X-100, 150 mM NaCl, 50 mM NaF, 2 mM Na-vanadate, 0.011 g ml^−1^ of β-glycerophosphate, 1× PhosSTOP phosphatase inhibitors, 1× cOmplete protease inhibitors) for 10 min on ice. Samples were clarified by centrifugation (14,000*g*, 15 min, 4 °C) and supernatants were transferred to new tubes. The protein concentration was measured using a Protein Assay Dye Reagent (BioRad catalog no. 5000006). The protein samples were subjected to electrophoretic separation on sodium dodecylsulfate–polyacrylamide gel electrophoresis and analyzed by standard western blotting techniques. In brief, proteins were transferred to nitrocellulose membranes (Amersham catalog no. 10600002), stained with 0.2% Ponceau solution (Serva, catalog no. 33427.01) to confirm equal loading. Membranes were blocked with 5% powdered milk in PBS-T (1× PBS, 0.1% Tween-20) for 1 h at room temperature, washed 3× for 10 min with PBS-T and incubated with primary antibodies (1:1,000 in PBS-T, 5% BSA) rotating overnight at 4 °C. The next day, membranes were washed 3× for 10 min with PBS-T and incubated with an HRP-conjugated anti-rabbit secondary antibody (1:10,000 in PBS-T, 5% milk) for 1 h at room temperature. Signals were detected by enhanced chemiluminescence (ECL), using the ECL Western Blotting Substrate (Promega, catalog no. W1015) or SuperSignal West Femto Substrate (Thermo Fisher Scientific, catalog no. 34095) for weaker signals. Western blot images were captured on film (GE Healthcare, catalog no. 28906835). A list of antibodies is provided in Supplementary Table 18.

### Co-immunoprecipitation

Worms expressing HA::RNP-6, RBM-39::mKate2 or both were harvested and proteins were extracted using the following standard protocol^74^. A solubilization buffer containing 0.5% NP40, 150 mM NaCl and 50 mM Tris, pH 7.4 supplemented with cOmplete Protease Inhibitor (Roche) and PhosSTOP (Roche) was used for immunoprecipitation. Flag immunoprecipitation was performed using Dynabeads Protein G (Thermo Fisher Scientific) and FLAG M2 mouse monoclonal antibody (Sigma-Aldrich), following the manufacturers’ protocols. Proteins were eluted from the beads by boiling with Laemmli buffer.

### Worm imaging

Analysis of worm reporters GFP::RNP-6, RBM-39::mKate2, mNeonGreen::EGL-8 and HLH-30::mNeonGreen was performed on a Zeiss Axioplan2 microscope (Axio Vision SE64, Rel.4.9.1) with a Zeiss AxioCam 506 CCD camera. Analysis of worm size was performed on a Leica stereo microscope (Leica M165 FC, LAS X) with Leica DFC3000G CCD. Fiji software (v.2.0.0/1.52p)^75^ was used for quantifying fluorescence intensity. For mNeonGreen::EGL-8 images, the head neuron region was selected for quantification. For HLH-30::mNeonGreen images, the nuclei of hypodermal cells were selected for quantification. For GFP::RNP-6 images, the whole worm was selected for quantification. To reduce bias, individual worms were randomly picked under a dissection microscope and imaged. At least 20 worms per genotype were picked for imaging and all the experiments were done 3× independently. Data from a representative experiment are shown in the figures for all the imaging panels.

### Transient knockdowns in HEK293FT cells (siRNA transfections)

Transient knockdowns were performed using a pool of four small interfering GENOME siRNAs (Horizon Discoveries) against PUF60, whereas an RLuc duplex siRNA that targets the *Rotylenchulus* *reniformis* luciferase gene (Horizon Discoveries) was used as control. In brief, HEK293FT cells were seeded in 12-well plates at 20% confluence and the following day transfected with 20 nM of the siRNA pool using Lipofectamine RNAiMAX (Thermo Fisher Scientific) according to the manufacturer’s instructions. Cells were harvested or fixed 72 h post-transfection and knockdown efficiency was verified by western blotting.

### Immunofluorescence and confocal microscopy in HEK293FT cells

Ιmmunofluorescence/confocal microscopy experiments and quantification of colocalization were performed as previously described^60^. In brief, cells were seeded on fibronectin-coated coverslips and treated as indicated in each experiment. After treatment, cells were fixed for 10 min at room temperature with 4% paraformaldehyde in PBS. Samples were washed/permeabilized with PBT solution (1× PBS-T), and blocked with BBT solution (1× PBS-T, 0.1% BSA). Staining was performed with the indicated primary antibodies in BBT (1:200 dilution) for 2 h at room temperature for mTOR and LAMP2 staining or overnight at 4 °C for TFE3 staining. Next, samples were washed 4× with BBT (15 min each), followed by incubation with appropriate highly cross-adsorbed, secondary fluorescent antibodies (1:200 in BBT) for 1 h at room temperature (Supplementary Table 18). Finally, nuclei were stained with DAPI and cells mounted on slides using Fluoromount-G (Invitrogen, catalog no. 00-4958-02). Images from single-channel captures are shown in grayscale. For the merged images, Alexa 488 is shown in green, TRITC in red and DAPI in blue. Images were captured using a ×40 objective lens on an SP8 Leica confocal microscope (Leica Application Suite X 3.5.7.23225). To quantify colocalization of mTOR signal with the lysosomal marker LAMP2, the Fiji software (v.2.1.0/1.53c)^75^ was used to define regions of interest corresponding to individual cells, excluding the nucleus. Fifty individual cells from five independent fields per condition were selected for the analysis. The Coloc2 plugin was used to calculate the Manders’ colocalization coefficient (MCC), using automatic Costes’ thresholding^76,77^. The MCC yields the fraction of the mTOR signal that overlaps with the LAMP2 signal. Subcellular localization of TFE3 was analyzed by scoring cells based on the signal distribution of TFE3, as shown in the example images in Fig. 5c. Signal was scored as nuclear (more TFE3 signal in the nucleus) or cytoplasmic (similar TFE3 signal between nucleus and cytoplasm). Cells from five independent fields, containing approximately 70 individual cells, were scored per condition for each experiment. Data from a representative experiment out of three independent replicates are shown in the figures for all confocal microscopy panels.

### Statistics and reproducibility

In all figure legends, ‘*n*’ denotes the number of independent experiments performed, whereas ‘*N*’ indicates the total number of animals analyzed in each condition. All statistical analyses were performed in GraphPad Prism (v.9.0.0 (86)). Asterisks denote corresponding statistical significance: ^*^P < 0.05; ^**^P < 0.01; ^***^P < 0.001; ^****^P < 0.0001. Data distribution was assumed to be normal but this was not formally tested. No statistical method was used to predetermine sample size but our sample sizes are similar to those reported in our previous publications^13,52,78,79^. No data were excluded from the analyses. At least three independent experiments for each assay were performed to verify the reproducibility of the findings (if there were two independent experiments, this was also noted in the figure legend). For the worm experiments, sample preparations and data collection were randomized. For lifespan experiments, all the genotypes and RNAi treatments were blinded. For cold tolerance, developmental rate, body area, infection, western blotting and imaging experiments, the genotypes were not blinded before assay because mutant worms have obvious phenotypes that revealed the sample identity (body size and developmental rate). However, worms were randomly picked and assigned to the different treatment conditions and the different conditions were assessed in random order. For RNA-seq experiments, the genotypes were not blinded before collecting samples. Once the RNA samples were ready, they were processed by staff at the Cologne Centre for Genomics in a blinded manner. For mammalian cell studies, no blinding was included in the data collection or analysis, because the method of quantification over multiple replicates and individual cells (for microscopy experiments) ensures unbiased processing.

### Reporting summary

Further information on research design is available in the Nature Research Reporting Summary linked to this article.

## Supplementary information

Supplementary Information Legends of Supplementary Tables and Video.

Reporting Summary

Supplementary Table 1 Supplementary table.

Supplementary Video 1 Supplementary video.

## Data Availability

All RNA-seq datasets generated and analyzed in the present study are available in the Gene Expression Omnibus datasets with the accession no. PRJNA757629. For protein alignment and phylogenetic analysis, all sequences are accessible through the UniProt database (https://www.uniprot.org) with the UniProt ID no. All other data are available as Source data files or from the corresponding author upon reasonable request.

## Source data

Source Data Fig. 1 Statistical source data.
Source Data Fig. 2 Statistical source data.
Source Data Fig. 3 Statistical source data.
Source Data Fig. 3 Unprocessed gels.
Source Data Fig. 4 Statistical source data.
Source Data Fig. 4 Unprocessed western blots.
Source Data Fig. 5 Statistical source data.
Source Data Fig. 5 Unprocessed western blots.
Source Data Extended Data Fig. 1 Statistical source data.
Source Data Extended Data Fig. 1 Unprocessed western blots.
Source Data Extended Data Fig. 2 Statistical source data.
Source Data Extended Data Fig. 3 Statistical source data.
Source Data Extended Data Fig. 3 Unprocessed western blots.
Source Data Extended Data Fig. 4 Statistical source data.
Source Data Extended Data Fig. 5 Statistical source data.
Source Data Extended Data Fig. 5 Unprocessed gels.
Source Data Extended Data Fig. 6 Statistical source data.
Source Data Extended Data Fig. 6 Unprocessed gels.
Source Data Extended Data Fig. 7 Statistical source data.
Source Data Extended Data Fig. 7 Unprocessed gels.
Source Data Extended Data Fig. 8 Statistical source data.
Source Data Extended Data Fig. 8 Unprocessed western blots.
Source Data Extended Data Fig. 9 Statistical source data.
Source Data Extended Data Fig. 9 Unprocessed western blots and gels.
