# Supplementary material for: Decreased spliceosome fidelity and egl-8 intron retention inhibit mTORC1 signaling to promote longevity
Source: Nat Aging. 2022 Sep 19;2(9):796–808. doi: 10.1038/s43587-022-00275-z (PMC10154236; doi:10.1038/s43587-022-00275-z)
Supplement: Supplementary file 1 — Legends of Supplementary Tables and Video. [file 43587_2022_275_MOESM1_ESM.pdf]

nature portfolio

https://doi.org/10.1038/s43587-022-00275-z

Supplementary information

Decreased spliceosome fidelity and *egl-8* intron retention inhibit mTORC1 signaling to promote longevity

In the format provided by the authors and unedited

Supplemental table legends

Supplementary Table 1. Alternative splicing analysis of RNAseq dataset from *rnp-6(G281D)* and *rnp-6i*. Events with Benjamini-Hochberg (BH)-adjusted P value below 0.05 were defined as significant.

Supplementary Table 2. Intron reads analysis of RNAseq dataset from *rnp-6(G281D)* and *rnp-6i*. Events P value below 0.001 were defined as significant.

Supplementary Table 3. circularRNA analysis of RNAseq dataset from *rnp-6(G281D)* and *rnp-6i*. Events with BH-adjusted P value below 0.05 were defined as significant.

Supplementary Table 4. Gene expression analysis of RNAseq dataset from *rnp-6(G281D)* and *rnp-6i*. Events with BH-adjusted P value below 0.05 were defined as significant.

Supplementary Table 5. Gene enrichment analysis of RNAseq dataset from *rnp-6(G281D)* and *rnp-6i*. P-values are calculated from Fisher's exact tests and adjusted with Bonferroni multiple hypothesis test. $P<0.05$ was defined as significant.

Supplementary Table 6. circularRNA analysis of RNAseq from *rbm-39(S294L)* suppressor datasets. Events with BH-adjusted P value below 0.05 were defined as significant.

Supplementary Table 7. Intron reads analysis of RNAseq from *rbm-39(S294L)* suppressor datasets. Events P value below 0.001 were defined as significant.

Supplementary Table 8. Alternative splicing analysis of RNAseq from *rbm-39(S294L)* suppressor datasets. Events with Benjamini-Hochberg (BH)-adjusted P value below 0.05 were defined as significant.

Supplementary Table 9. Gene expression analysis of RNAseq from *rbm-39(S294L)* suppressor datasets. Events with BH-adjusted P value below 0.05 were defined as significant.

Supplementary Table 10. Alternative splicing analysis of RNAseq from *rnp-6(E161K)* suppressor datasets. Events with Benjamini-Hochberg (BH)-adjusted P value below 0.05 were defined as significant.

Supplementary Table 11. Intron reads analysis of RNAseq from *rnp-6(E161K)* suppressor datasets. Events P value below 0.001 were defined as significant.

Supplementary Table 12. circularRNA analysis of RNAseq from *rnp-6(E161K)* suppressor datasets. Events with BH-adjusted P value below 0.05 were defined as significant.

Supplementary Table 13. Gene expression analysis of RNAseq from *rnp-6(E161K)* suppressor datasets. Events with BH-adjusted P value below 0.05 were defined as significant.

Supplementary Table 14. List of intron retention events used for lifespan screen.

Supplementary Table 15. Information of primers used in this study.

Supplementary Table 16. Lifespan results used in this study. P values were calculated using the log-rank (Mantel-Cox) analysis method.

Supplementary Table 17. Infection experiments results used in this study. P values were calculated using the log-rank (Mantel-Cox) analysis method.

Supplementary Table 18. Antibodies, chemical reagents, bacterial strains, worm strains and software used in this study.

Supplementary video legend

Supplementary video 1. RBM-39(S294L) puncta are dynamic.

Time lapse imaging of mNeonGreen tagged RBM-39(S294L) puncta. White arrows indicate two highly dynamic events. scale bar, 20 μm.
