# Supplementary material for: Decreased spliceosome fidelity and egl-8 intron retention inhibit mTORC1 signaling to promote longevity
Source: Nat Aging. 2022 Sep 19;2(9):796–808. doi: 10.1038/s43587-022-00275-z (PMC10154236; doi:10.1038/s43587-022-00275-z)
Supplement: Supplementary file 2 — Reporting Summary [file 43587_2022_275_MOESM2_ESM.pdf]

Corresponding author(s): Adam Antebi
Last updated by author(s): Jun 19, 2022

Reporting Summary

Nature Portfolio wishes to improve the reproducibility of the work that we publish. This form provides structure for consistency and transparency in reporting. For further information on Nature Portfolio policies, see our Editorial Policies and the Editorial Policy Checklist.

Statistics

For all statistical analyses, confirm that the following items are present in the figure legend, table legend, main text, or Methods section.

n/a	Confirmed	
☐	☒	The exact sample size (*n*) for each experimental group/condition, given as a discrete number and unit of measurement
☐	☒	A statement on whether measurements were taken from distinct samples or whether the same sample was measured repeatedly
☐	☒	The statistical test(s) used AND whether they are one- or two-sided<br>*Only common tests should be described solely by name; describe more complex techniques in the Methods section.*
☐	☒	A description of all covariates tested
☐	☒	A description of any assumptions or corrections, such as tests of normality and adjustment for multiple comparisons
☐	☒	A full description of the statistical parameters including central tendency (e.g. means) or other basic estimates (e.g. regression coefficient) AND variation (e.g. standard deviation) or associated estimates of uncertainty (e.g. confidence intervals)
☐	☒	For null hypothesis testing, the test statistic (e.g. F, t, r) with confidence intervals, effect sizes, degrees of freedom and P value noted<br>*Give P values as exact values whenever suitable.*
☒	☐	For Bayesian analysis, information on the choice of priors and Markov chain Monte Carlo settings
☒	☐	For hierarchical and complex designs, identification of the appropriate level for tests and full reporting of outcomes
☐	☒	Estimates of effect sizes (e.g. Cohen's *d*, Pearson's *r*), indicating how they were calculated

Our web collection on statistics for biologists contains articles on many of the points above.

Software and code

Policy information about availability of computer code

Data collection

No code for data collection was generated in this study.

Commercial software packages from other developers used in this study are:
Confocal microscopy: Leica Application Suite X 3.5.7.23225;
Zeiss microscopy: Axio Vision SE64Rel.4.9.1
Leica M165 FC:LAS X
DNA Gel imaging and Western blotting: BioRad ChemiDoc MP, Image Lab 6.1

Data analysis

No code for data analysis was generated in this study.

Commercial software packages from other developers used in this study are:
Statistical analysis and graph preparation: GraphPad Prism 9.0.0 (86); https://www.graphpad.com/
Quantification of colocalization: ImageJ/Fiji (Version 2.0.0/1.52p, 2.1.0/1.53c); https://imagej.net/software/fiji/

RNAseq analysis: Reads alignment was performed with the Hisat version 2.0.4 64. Differentially expressed genes between different samples were identified using the Stringtie (version 1.3.0), followed by Cufflinks (version 2.2). The enrichment visualization was performed with WormCat 2.0. Splicing analysis was performed with SAJR pipeline. For intron retention analysis, Bedtools coverage (version 2.29.0) was used to count intron and total gene expression. IBB (version 20.06; R version 4.0.3) was used to calculate differential intron expression. DCC/CircTest (Version: 0.1.0) pipeline was performed to quantify Circular RNAs expression. T-coffee (Version_11.00, https://tcoffee.crg.eu) was used to align RNP-6, RBM-39 and their homologs from different species. Phylogeny.fr (http://www.phylogeny.fr/index.cgi) was used to perform phylogenetic analysis. Flaski (Version 2.0.0) (DOI: 10.5281/zenodo.5254193) was used to generate row Z-score heatmaps.

For manuscripts utilizing custom algorithms or software that are central to the research but not yet described in published literature, software must be made available to editors and reviewers. We strongly encourage code deposition in a community repository (e.g. GitHub). See the Nature Portfolio guidelines for submitting code & software for further information.

Data

Policy information about availability of data

All manuscripts must include a data availability statement. This statement should provide the following information, where applicable:

- Accession codes, unique identifiers, or web links for publicly available datasets
- A description of any restrictions on data availability
- For clinical datasets or third party data, please ensure that the statement adheres to our policy

There is no restriction on data availability. Source data are provided with this paper. All RNA-seq raw data are available in the GEO datasets: PRJNA757629. For the protein alignment and phylogenetic analysis, all the sequences are accessible through Uniprot database (https://www.uniprot.org) with the UniProt ID number.

Human research participants

Policy information about studies involving human research participants and Sex and Gender in Research.

Reporting on sex and gender	N/A
Population characteristics	N/A
Recruitment	N/A
Ethics oversight	N/A

Note that full information on the approval of the study protocol must also be provided in the manuscript.

Field-specific reporting

Please select the one below that is the best fit for your research. If you are not sure, read the appropriate sections before making your selection.

☒ Life sciences ☐ Behavioural & social sciences ☐ Ecological, evolutionary & environmental sciences

For a reference copy of the document with all sections, see nature.com/documents/nr-reporting-summary-flat.pdf

Life sciences study design

All studies must disclose on these points even when the disclosure is negative.

Sample size

No statistical methods were used for sample size determination. Exact sample sizes are indicated in the corresponding figure legends and supplementary tables.
For RNAseq experiments, sample sizes were chosen based on our prior studies (Tharyan et al., Nat Metab. 2020, Kew et al., Elife . 2020). Sample sizes for cold tolerance, developmental rate, body area, infection killing assay, western blot, RT-PCR, life span and quantification of fluorescence reporters in C. elegans were determined according to our laboratory experience and other studies using these assays (Kew et al., Elife. 2020, Tiku et al., Nat Commun. 2017, Irazoqui et al., PLoS Pathog. 2010). For colocalization and western blotting assays in mammalian cells, sample sizes were determined in accordance with standard practices in the field and based on our long-standing experience in this type of experimental approaches (e.g., PMIDs 26868506, 33497611, 33974911). As indicated in the methods section, for colocalization studies, 50 individual cells from 5 randomly selected fields were analyzed per condition.

Data exclusions

No data were excluded from the analyses.

Replication	At least three independent experiments for each assay were performed to verify the reproducibility of the findings (if there were two independent experiments, this was also noticed in the figure legend).
Randomization	For cold tolerance, developmental rate, body area, infection killing assay, western blot, RT-PCR, life span and imaging experiments, young adult stage hermaphrodites were randomly picked from our maintenance plates let them lay eggs for 4 hours. When the progeny reach young adult stage, they were randomly picked and assigned to the different treatment conditions. The different conditions were assessed in random order. For RNAseq experiments, worms were synchronized by egg laying. ~300 day 1 adult stage worms were randomly picked from our maintenance plates let them lay eggs for 6 hours. Afterwards, the worms are discarded by washing with M9 buffer and eggs were harvested and randomly assigned to the different treatment conditions. When worms reached young adult stage, they were collected, lysed and used for RNA preparation in random order.<br>Sample randomization was not performed for the mammalian cell experiments described in this study, as the order of analysis does not influence the experimental outcomes.
Blinding	For life span experiments, all the genotypes and RNAi treatments were blinded. For cold tolerance, developmental rate, body area, infection, western blot and imaging experiments, the genotypes were not blinded before assay as mutants worms have obvious phenotypes that revealed the sample identity (body size and developmental rate). However, worms were randomly picked and assigned to the different treatment conditions and the different conditions were assessed in random order. Moreover, all the critical experiments were repeated independently by at least 3 times. For RNAseq experiments, the genotypes were not blinded before collecting samples. Once the RNA samples were ready, they were processed by staff of Cologne Center for Genomics (CCG) in a blinded manner.<br>For mammalian cell studies, no blinding was included in the data collection or analysis, as the method of quantification over multiple replicates and individual cells (for microscopy experiments) ensures unbiased processing.

Reporting for specific materials, systems and methods

We require information from authors about some types of materials, experimental systems and methods used in many studies. Here, indicate whether each material, system or method listed is relevant to your study. If you are not sure if a list item applies to your research, read the appropriate section before selecting a response.

Materials & experimental systems

n/a	Involved in the study
☐	☒ Antibodies
☐	☒ Eukaryotic cell lines
☒	☐ Palaeontology and archaeology
☐	☒ Animals and other organisms
☒	☐ Clinical data
☒	☐ Dual use research of concern

Methods

n/a	Involved in the study
☒	☐ ChIP-seq
☒	☐ Flow cytometry
☒	☐ MRI-based neuroimaging

Antibodies

Antibodies used	Rat anti-HA Roche #3F10 RRID:AB_2314622 1:2000<br>Rabbit anti-Phospho-AMPKα (Thr172) CST #2535 RRID:AB_331250 1:2000<br>Rabbit anti-RFP ThermoFisher #R10367 RRID:AB_10563941 1:2000<br>Mouse anti-beta Actin Abcam #ab8224 RRID:AB_449644 1:5000<br>Mouse anti-FLAG Sigma-Aldrich #M2 RRID:AB_262044 1:2000<br>Anti-Mouse HRP ThermoFisher #G-21040 RRID: AB_2536527 1:5000<br>Anti-Rabbit HRP ThermoFisher #G-21234 RRID: AB_2536530 1:5000<br>Anti-Rat HRP GE Healthcare #NA935 RRID: AB_772207 1:5000<br>Rabbit anti-pTFEB (S211) CST #37681 RRID:AB_2799117 1:1000<br>Rabbit anti-TFEB CST #4240 RRID:AB_11220225 1:1000<br>Rabbit anti-pS6K (T389) CST #97596 RRID:AB_2800283 1:1000<br>Rabbit anti-S6K CST #9202 RRID:AB_331676 1:1000<br>Rabbit anti-RAPTOR CST #2280 RRID:AB_561245 1:1000<br>Rabbit anti-mTOR CST #2983 RRID:AB_2105622 1:1000 (WB) 1:200 (IF)<br>Rabbit anti-GAPDH CST #2118 RRID:AB_561053 1:1000<br>Rabbit anti-pAKT (S473) CST #9271 RRID:AB_329825 1:1000<br>Rabbit anti-AKT CST #9272 RRID:AB_329827 1:1000<br>Rabbit anti-RICTOR CST #2114 RRID:AB_2179963 1:1000<br>Rabbit anti-TFE3 CST #14779 RRID:AB_2687582 1:200 (IF)<br>Mouse anti-LAMP2 DSHB #H4B4 RRID:AB_528129 1:200 (IF)<br>Rabbit anti-PUF60 Thermo Fisher Scientific PA5-21411 RRID:AB_11154782 1:1000<br>Anti-Rabbit HRP Jackson ImmunoResearch #711-035-152 RRID:AB_10015282 1:10000<br>Anti-Rabbit Alexa Fluor 488 Jackson ImmunoResearch #711-545-152 RRID:AB_2313584 1:200 (IF)<br>Anti-Mouse Rhodamine (TRITC) Jackson ImmunoResearch #715-025-150 RRID:AB_2340766 1:200 (IF)
Validation	All the antibodies used in this study are commercially available and the validations were done by manufacturer and supported by the publications indicated in the manufacturer's website.<br><br>*Rat anti-HA Roche #3F10 RRID:AB_2314622 1:2000 The antibody was used according to the manufacturer's instructions for

western blot in C. elegans. References: PMID: 28916755.

*Rabbit anti-Phospho-AMPKα (Thr172) CST #2535 RRID:AB_331250 1:2000 This antibody has been validated for western blot in C. elegans in previous publication: PMID: 31411562.

*Rabbit anti-RFP ThermoFisher #R10367 RRID:AB_10563941 1:2000 This antibody has been validated for western blot in C. elegans in previous publication: PMID: 32356725.

*Mouse anti-beta Actin Abcam #ab8224 RRID:AB_449644 1:5000 This antibody has been validated for western blot in C. elegans in our previous publication: PMID: 32538777.

*Mouse anti-FLAG Sigma-Aldrich #M2 RRID:AB_262044 1:2000 This antibody has been validated for western blot in C. elegans in previous publication: PMID: 33514673.

*Rabbit anti-pTFEB (S211) CST #37681 RRID:AB_2799117 1:1000 According to the manufacturer and supported by previous studies, this antibody has been validated for western blot HEK293 cell line. References: PMID: 34253722, PMID: 34405859.

*Rabbit anti-TFEB CST #4240 RRID:AB_11220225 1:1000 According to the manufacturer and supported by previous studies, this antibody has been validated for western blot HEK293 cell line. References: PMID: 34405859, PMID: 27278822.

*Rabbit anti-pS6K (T389) CST #97596 RRID:AB_2800283 1:1000 According to the manufacturer and supported by previous studies, this antibody has been validated for western blot HEK293 cell line. References: PMID: 30837833, PMID: 33253182.

*Rabbit anti-S6K CST #9202 RRID:AB_331676 1:1000 According to the manufacturer and supported by previous studies, this antibody has been validated for western blot HEK293 cell line. References: PMID: 28670736, PMID: 30753671.

*Rabbit anti-RAPTOR CST #2280 RRID:AB_561245 1:1000 According to the manufacturer and supported by previous studies, this antibody has been validated for western blot HEK293 cell line. References: PMID: 28112156, PMID: 31112131.

*Rabbit anti-mTOR CST #2983 RRID:AB_2105622 1:1000 According to the manufacturer and supported by previous studies, this antibody has been validated for western blot HEK293 cell line. References: PMID: 31270333, PMID: 30753671.

*Rabbit anti-GAPDH CST #2118 RRID:AB_561053 1:1000 According to the manufacturer and supported by previous studies, this antibody has been validated for western blot HEK293 cell line. References: PMID: 34944949, PMID: 22676960.

*Rabbit anti-pAKT (S473) CST #9271 RRID:AB_329825 1:1000 According to the manufacturer and supported by previous studies, this antibody has been validated for western blot HEK293 cell line. References: PMID: 26094770, PMID: 24966332.

*Rabbit anti-AKT CST #9272 RRID:AB_329827 1:1000 According to the manufacturer and supported by previous studies, this antibody has been validated for western blot HEK293 cell line. References: PMID: 20003239, PMID: 27274457.

*Rabbit anti-RICTOR CST #2114 RRID:AB_2179963 1:1000 According to the manufacturer and supported by previous studies, this antibody has been validated for western blot HEK293 cell line. References: PMID: 34404770, PMID: 30068931

*Rabbit anti-TFE3 CST #14779 RRID:AB_2687582 1:1000 According to the manufacturer and supported by previous studies, this antibody has been validated for western blot HEK293 cell line. References: PMID: 31733992.

*Mouse anti-LAMP2 DSHB #H4B4 RRID:AB_528129 1:1000 According to the manufacturer and supported by previous studies, this antibody has been validated for western blot HEK293 cell line. References: PMID: 27994678, PMID: 22767497

*Rabbit anti-PUF60 Thermo Fisher Scientific #PA5-21411 RRID:AB_11154782 1:1000 This antibody has been validated by manufacturer in different cell lines. It is also validated in HEK293 cell line with this study by siRNA experiment.

*Anti-Mouse HRP ThermoFisher #G-21040 RRID: AB_2536527 1:5000 (worm), 1:10000 (HEK293)
*Anti-Rabbit HRP ThermoFisher #G-21234 RRID: AB_2536530 1:5000 (worm), 1:10000(HEK293)
*Anti-Rat HRP GE Healthcare #NA935 RRID: AB_772207 1:5000
*Anti-Rabbit HRP Jackson ImmunoResearch #711-035-152 RRID:AB_10015282 1:10000
*Anti-Rabbit Alexa Fluor 488 Jackson ImmunoResearch #711-545-152 RRID:AB_2313584 1:200 (IF)
*Anti-Mouse Rhodamine (TRITC) Jackson ImmunoResearch #715-025-150 RRID:AB_2340766 1:200 (IF)

Eukaryotic cell lines

Policy information about cell lines and Sex and Gender in Research

Cell line source(s)	The HEK293FT cells were purchased from Invitrogen before the initiation of the project.
Authentication	The identity of the HEK293FT cells was validated by the Multiplex human Cell Line Authentication test (Multiplexion GmbH), which uses a single nucleotide polymorphism (SNP) typing approach, and was performed as described at www.multiplexion.de.
Mycoplasma contamination	Cell lines were regularly tested for Mycoplasma contamination using a PCR-based approach and were confirmed to be Mycoplasma-free.
Commonly misidentified lines (See ICLAC register)	no commonly misidentified cell lines were used in this study.

Animals and other research organisms

Policy information about studies involving animals; ARRIVE guidelines recommended for reporting animal research, and Sex and Gender in Research

Laboratory animals	Caenorhabditis elegans strains were used in this study. The young adult stage hermaphrodites of all strains were used in experiments.<br>C. elegans: wild type CGC Strain: N2<br>C. elegans: rnp-6(dh1127). rnp-6(G281D) AA lab Strain: AA4548<br>C. elegans: rbm-39(syb1074). rbm-39(S294L).Outcrossed PHX1074 to N2-AA AA lab Strain: AA4924<br>C. elegans: rnp-6(dh1127);rbm-39(syb1074) AA lab Strain: AA4925<br>C. elegans: rnp-6(gk670228). rnp-6(E161K). Outcrossed VC40508 to N2-AA AA lab Strain: AA4961<br>C. elegans: rnp-6(dh1187). rnp-6(E161K,G281D). AA lab Strain: AA4958<br>C. elegans: rnp-6(dh1188). HA::rnp-6(wt) AA lab Strain: AA4624<br>C. elegans: rnp-6(dh1145).HA::rnp-6(G281D) AA lab Strain: AA4641<br>C. elegans: rnp-6(syb645). GFP::rnp-6(wt). Outcrossed PHX645 to N2-AA AA lab Strain: AA4824<br>C. elegans: rnp-6(syb626).GFP::rnp-6(G281D). Outcrossed PHX626 to N2-AA AA lab Strain: AA4841<br>C. elegans: rbm-39(syb1527). rbm-39(wt)::mKate2.Outcrossed PHX1527 to N2-AA AA lab Strain: PHX1527<br>C. elegans: rbm-39(syb1545). rbm-39(S294L)::mKate2.Outcrossed PHX1545 to N2-AA AA lab Strain: PHX1545<br>C. elegans: rnp-6(syb645);rbm-39(syb1527). AA lab Strain: AA5053<br>C. elegans: rnp-6(syb645);rbm-39(syb1545). AA lab Strain: AA5054<br>C. elegans: rnp-6(syb626);rbm-39(syb1527). AA lab Strain: AA5055<br>C. elegans: rnp-6(syb626);rbm-39(syb1545). AA lab Strain: AA5056<br>C. elegans: egl-8(n488). Outcrossed MT1083 to N2-AA AA lab Strain: AA5261<br>C. elegans: rnp-6(dh1127);egl-8(n488). AA lab Strain: AA5260<br>C. elegans: egl-8(syb3661). mNeonGreen::egl-8. Outcrossed to N2-AA AA lab Strain: AA5274<br>C. elegans: rnp-6(dh1127);egl-8(syb3661). AA lab Strain: AA5272<br>C. elegans: egl-8(syb4850). egl-8 intron 8 3' splicing site editing. AA lab Strain: PHX4850<br>C. elegans: raga-1(ok701).Outcrossed VC533 to N2-AA AA lab Strain: AA3776<br>C. elegans: rnp-6(dh1127);raga-1(ok701) AA lab Strain: AA4995<br>C. elegans: daf-2(e1370). Outcrossed CB1370 to N2-AA AA lab Strain: CB1370<br>C. elegans: rnp-6(dh1127);daf-2(e1370) AA lab Strain: AA4642<br>C. elegans: daf-16(mu86). Outcrossed CF1038 to N2-AA AA lab Strain: CF1038<br>C. elegans: rnp-6(dh1127);daf-16(mu86) AA lab Strain: AA4644<br>C. elegans: eat-2(ad465). Outcrossed to N2-AA AA lab Strain: DA465<br>C. elegans: rnp-6(dh1127);eat-2(ad465) AA lab Strain: AA4646<br>C. elegans: N2, dhEx1132[rnp-6p::gfp::rnp-6, myo-2p::GFP] AA lab Strain:<br>C. elegans: rnp-6(dh1127), dhEx1132[rnp-6p::gfp::rnp-6, myo-2p::GFP] AA lab Strain:<br>C. elegans: N2, dhEx1208[unc-17p::gfp::egl-8a cDNA, myo-3p::mCherry] AA lab Strain: AA5238<br>C. elegans: rnp-6(dh1127), dhEx1208[unc-17p::gfp::egl-8a cDNA, myo-3p::mCherry] AA lab Strain: AA5239<br>C. elegans: N2, dhEx1170[rgef-1p::gfp::rnp-6b cDNA, myo-3p::mCherry] AA lab Strain: AA4752<br>C. elegans: rnp-6(dh1127), dhEx1170[rgef-1p::gfp::rnp-6b cDNA, myo-3p::mCherry] AA lab Strain: AA5232<br>C. elegans: N2, dhEx1139[rnp-6p::gfp::rnp-6b cDNA(wt), myo-3p::mCherry] AA lab Strain: AA4632<br>C. elegans: rnp-6(dh1127), dhEx1139[rnp-6p::gfp::rnp-6b cDNA(wt), myo-3p::mCherry] AA lab Strain: AA4663<br>C. elegans: N2, dhEx1147[rnp-6p::gfp::rnp-6b cDNA(G281D), myo-3p::mCherry] AA lab Strain: AA4657<br>C. elegans: rnp-6(dh1127), dhEx1147[rnp-6p::gfp::rnp-6b cDNA(G281D), myo-3p::mCherry] AA lab Strain: AA4910
Wild animals	This study did not involve wild animals.
Reporting on sex	Sex was not considered in the study design. All the C. elegans strains used were hermaphrodite.
Field-collected samples	This study did not involve field-collected samples.
Ethics oversight	No ethical approval was required.

Note that full information on the approval of the study protocol must also be provided in the manuscript.
