# Supplementary figures and images for: Decreased spliceosome fidelity and egl-8 intron retention inhibit mTORC1 signaling to promote longevity
Source: Nat Aging. 2022 Sep 19;2(9):796–808. doi: 10.1038/s43587-022-00275-z (PMC10154236; doi:10.1038/s43587-022-00275-z)

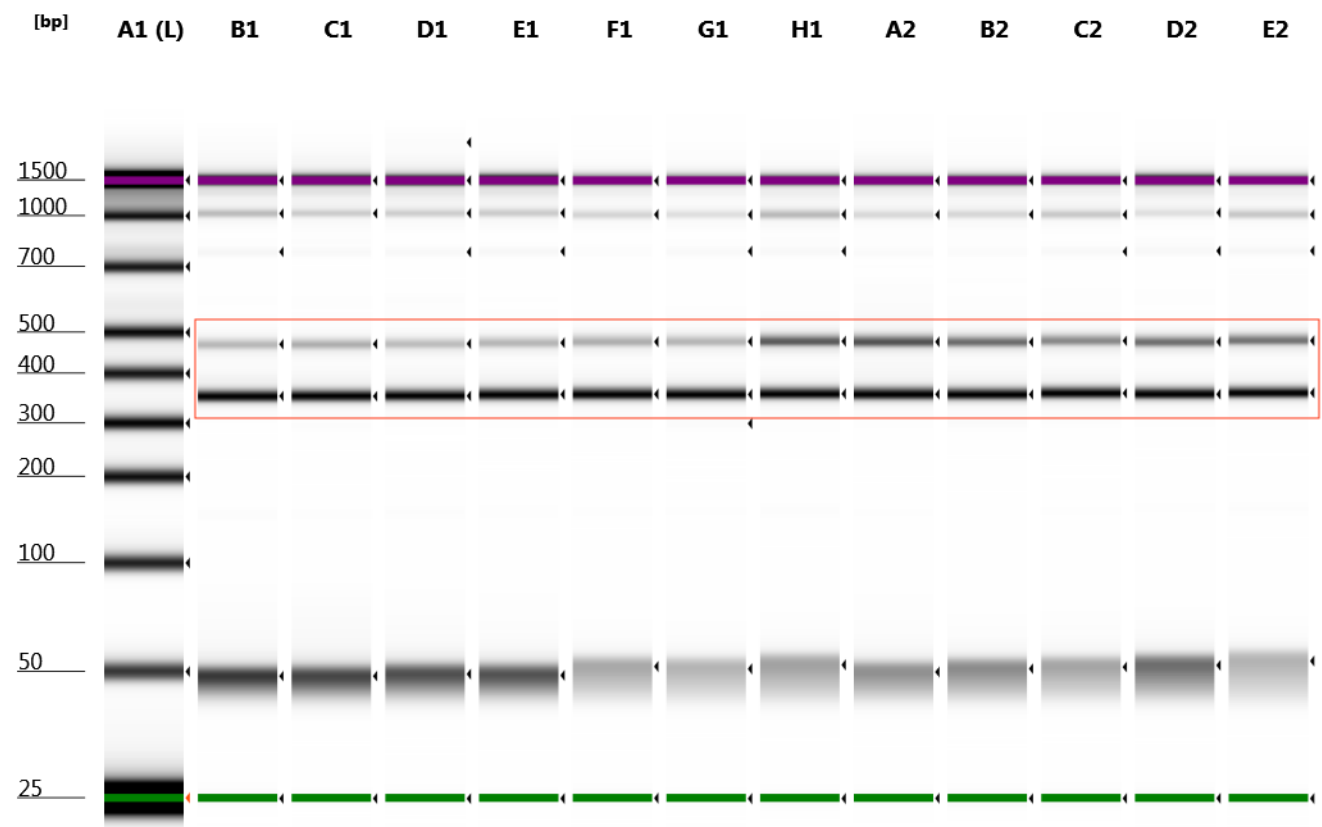

Supplement: Source Data Fig. 3 — Unprocessed gels. [file 43587_2022_275_MOESM8_ESM.pdf]

**Fig.4e**  
**Anti p-AMPK**

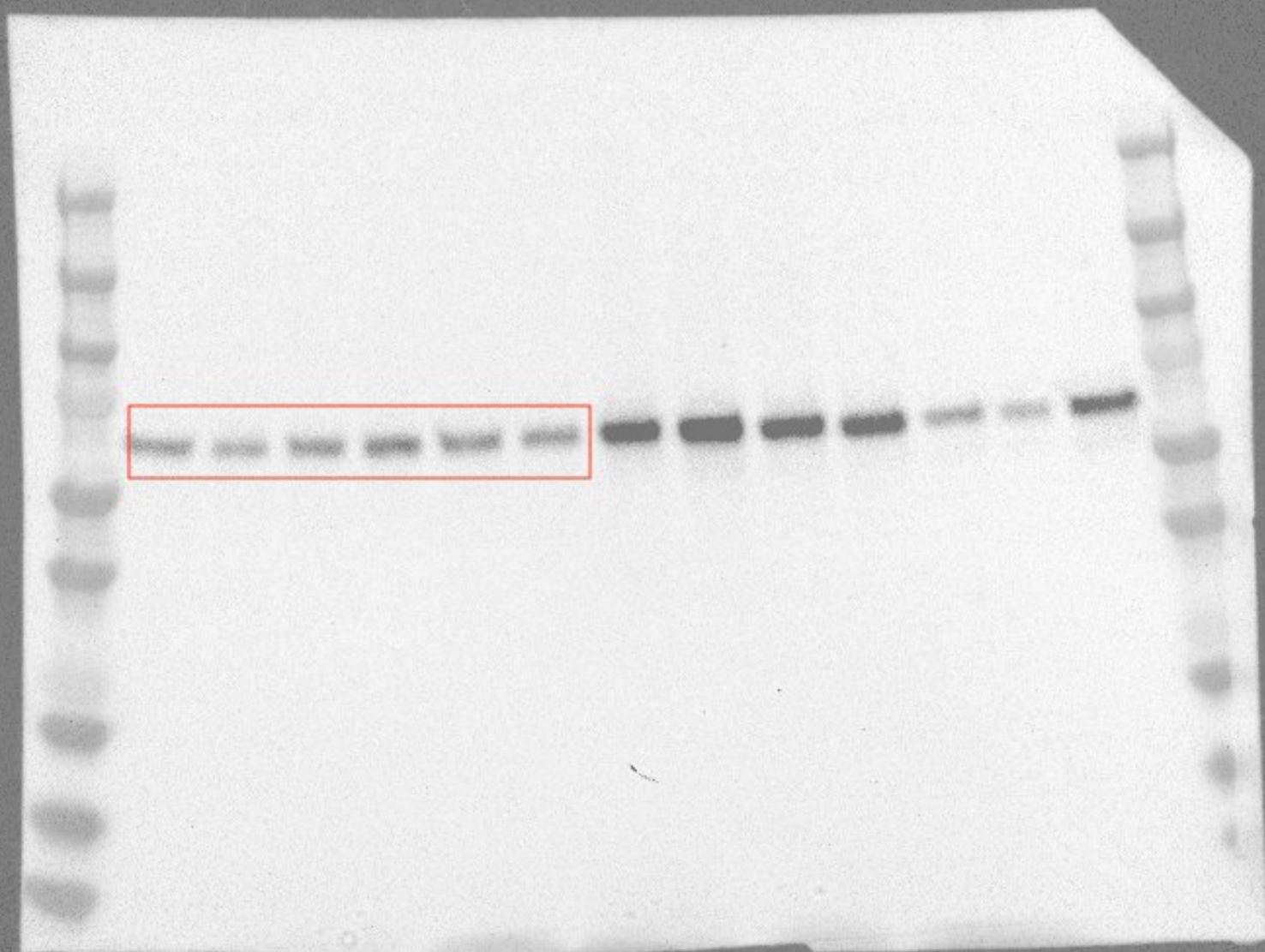

**Fig.4e**  
**Anti beta actin**

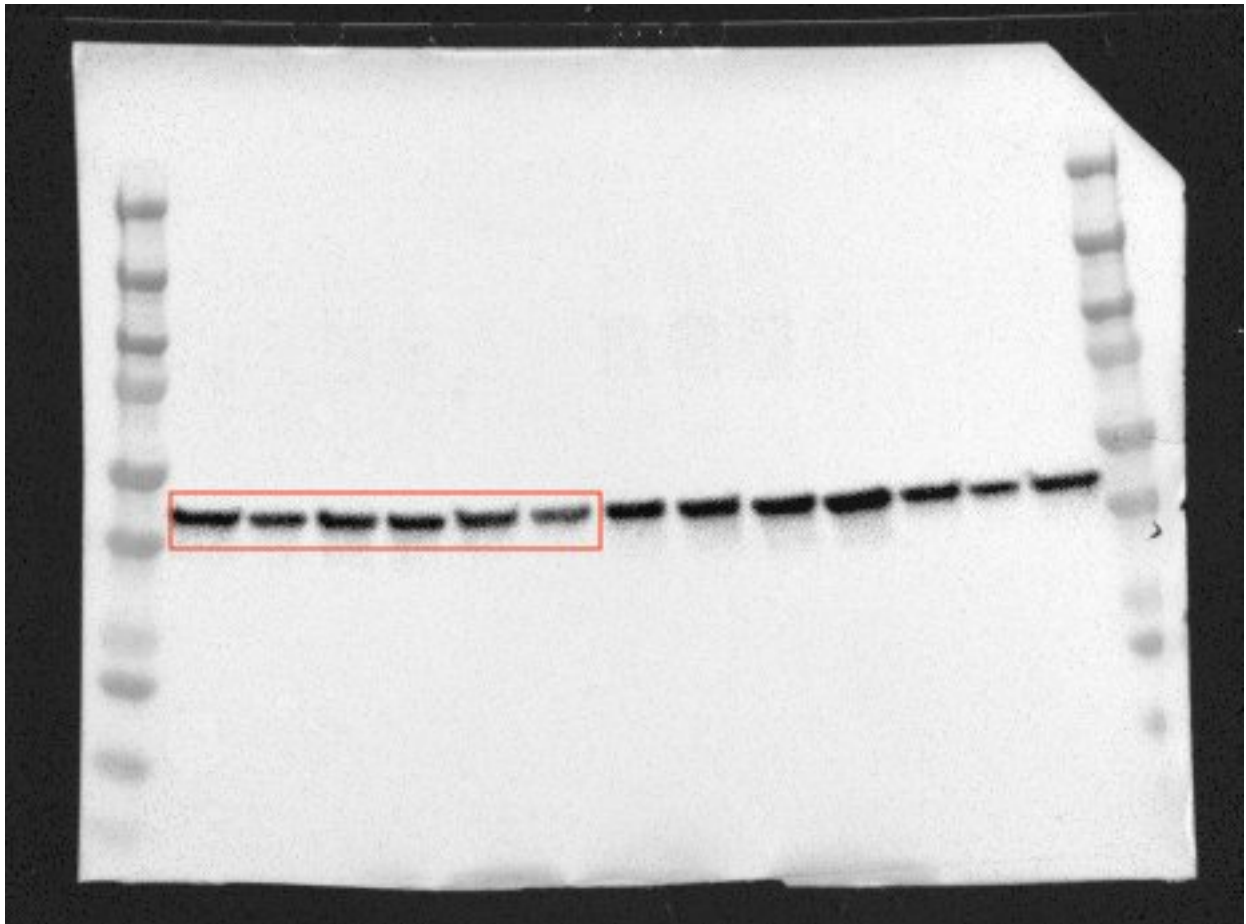

Supplement: Source Data Fig. 4 — Unprocessed western blots. [file 43587_2022_275_MOESM10_ESM.pdf]

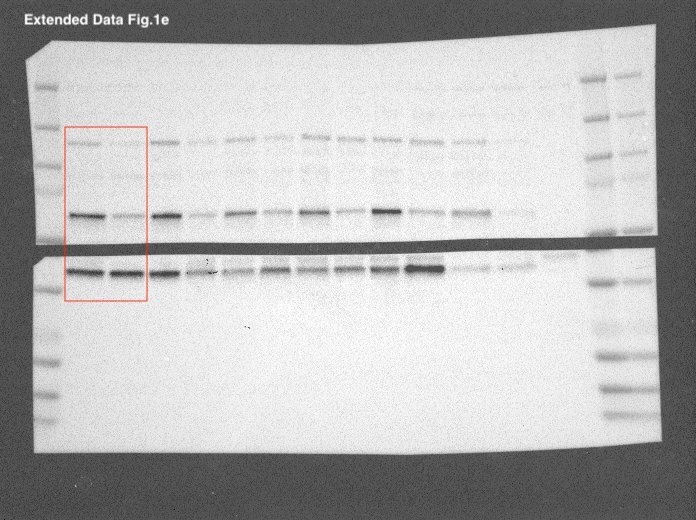

Supplement: Source Data Extended Data Fig. 1 — Unprocessed western blots. [file 43587_2022_275_MOESM14_ESM.jpg]

A1 B1 C1 D1 E1 F1 A2 B2 C2 D2 E2 F2

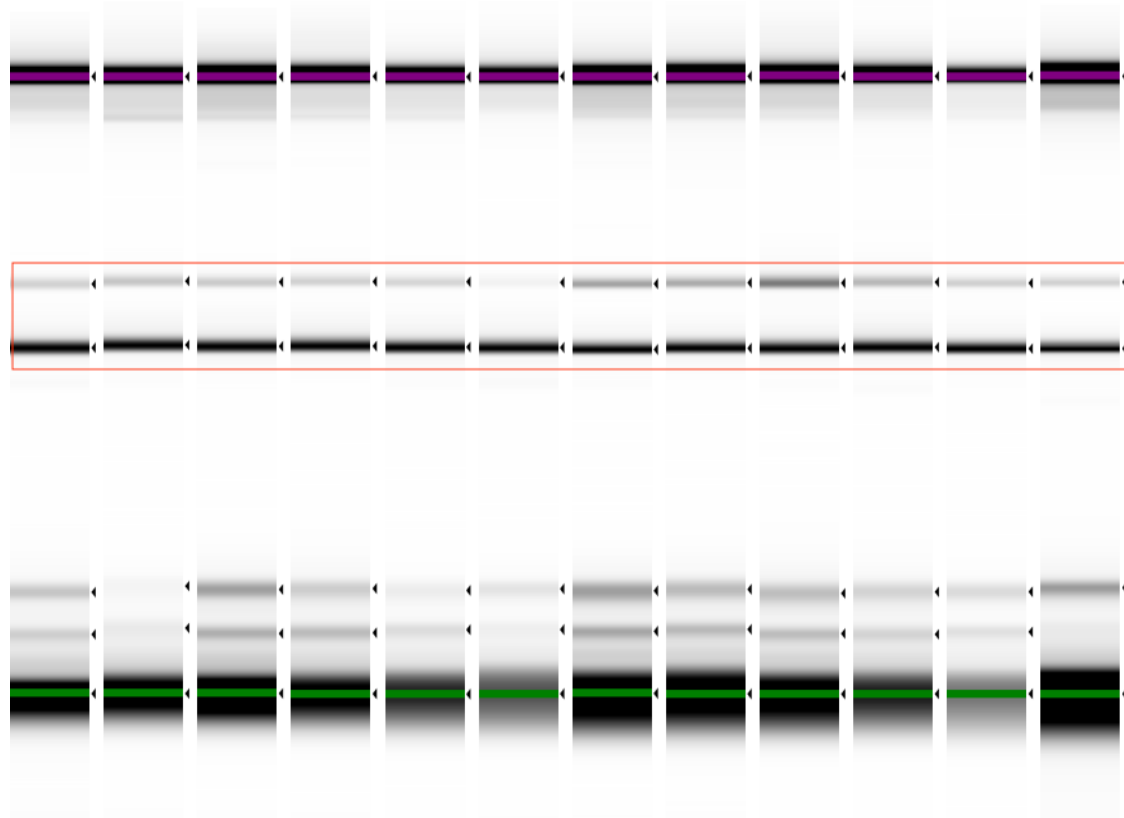

Supplement: Source Data Extended Data Fig. 5 — Unprocessed gels. [file 43587_2022_275_MOESM20_ESM.pdf]

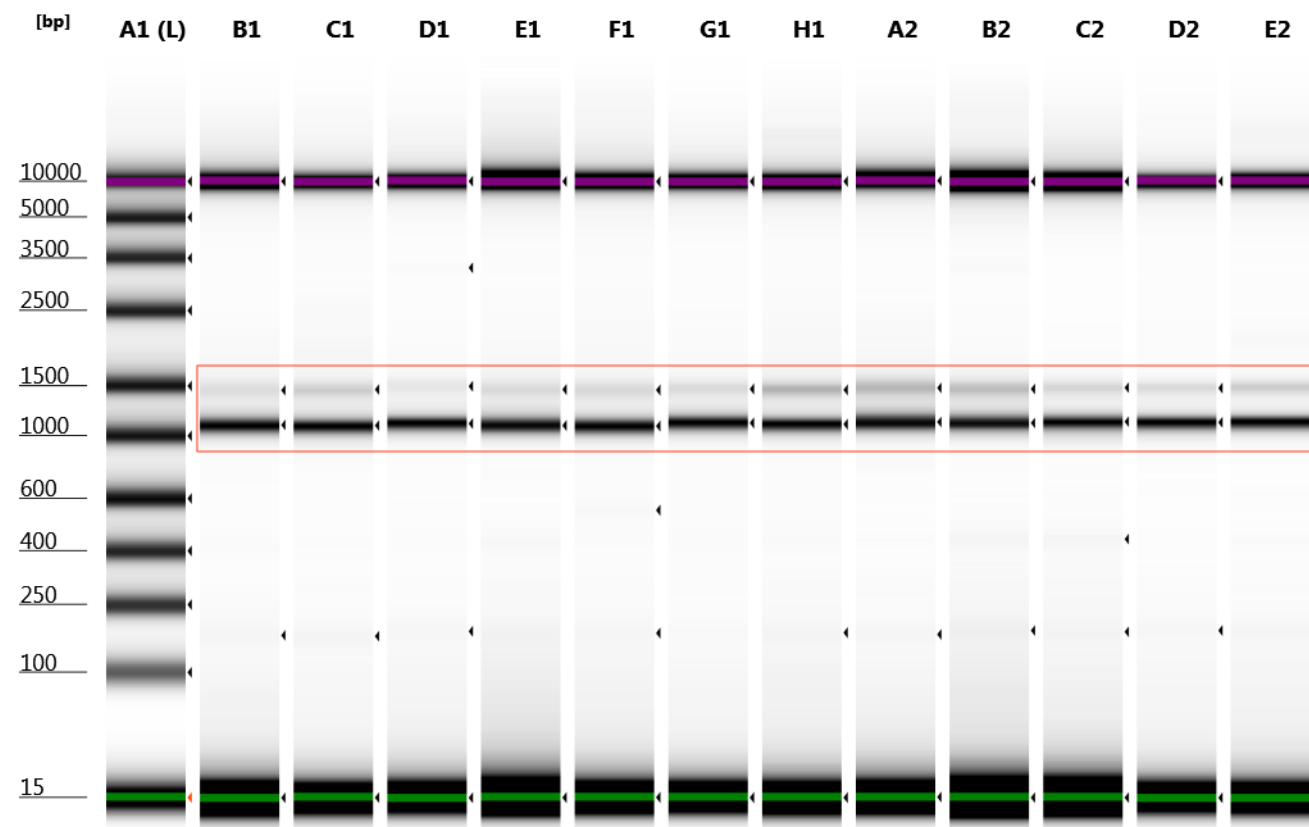

Supplement: Source Data Extended Data Fig. 6 — Unprocessed gels. [file 43587_2022_275_MOESM22_ESM.pdf]

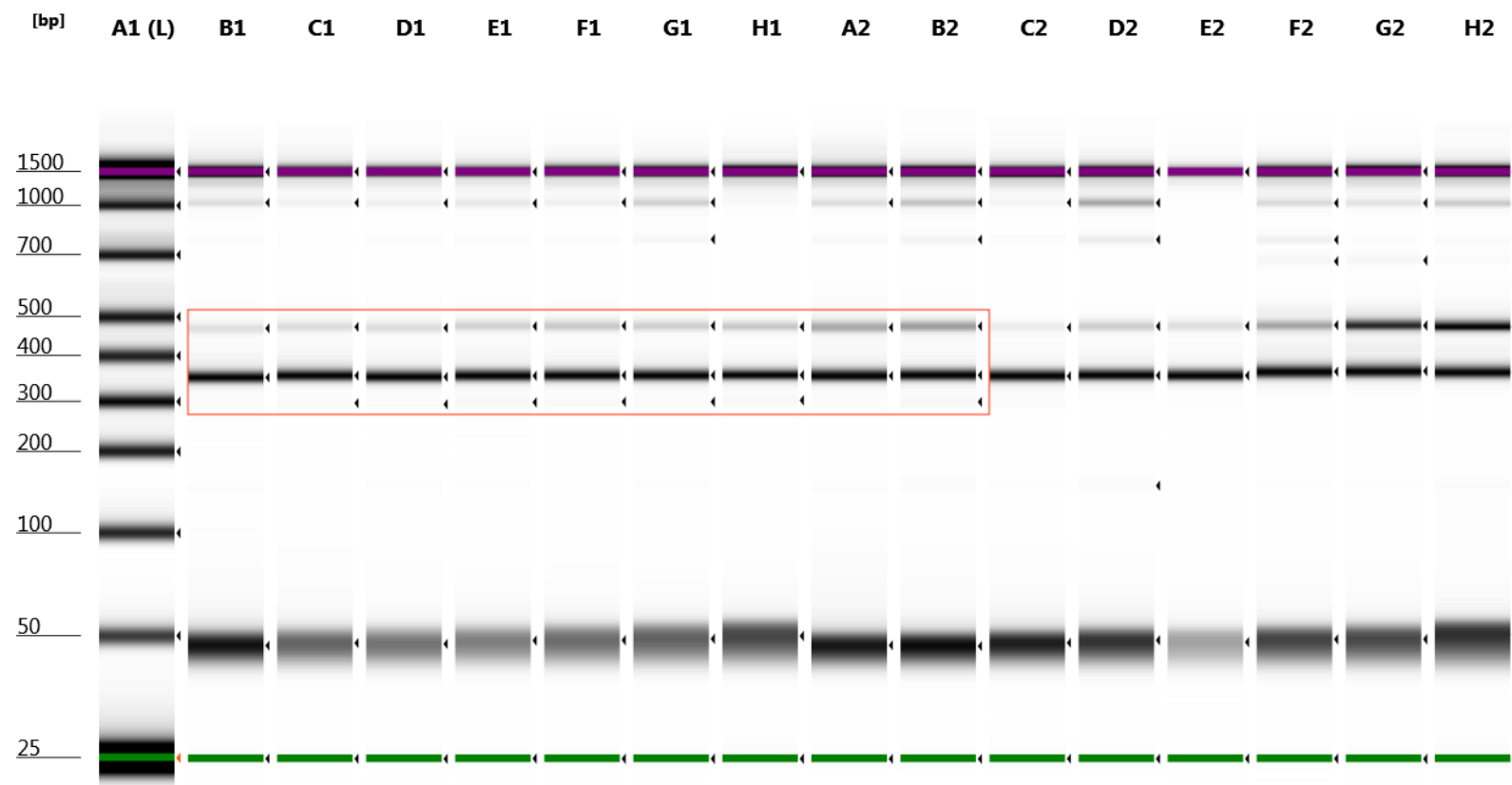

Supplement: Source Data Extended Data Fig. 7 — Unprocessed gels. [file 43587_2022_275_MOESM24_ESM.pdf]

**Extended Data Fig.8g**  
**Anti p-AMPK**

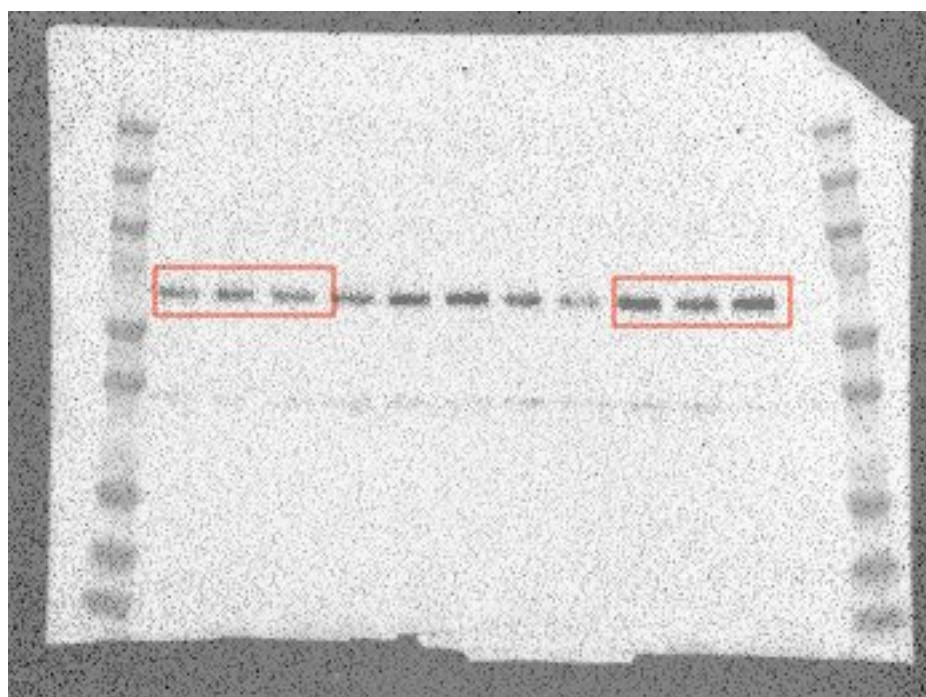

Extended Data Fig.8g  
Anti beta actin

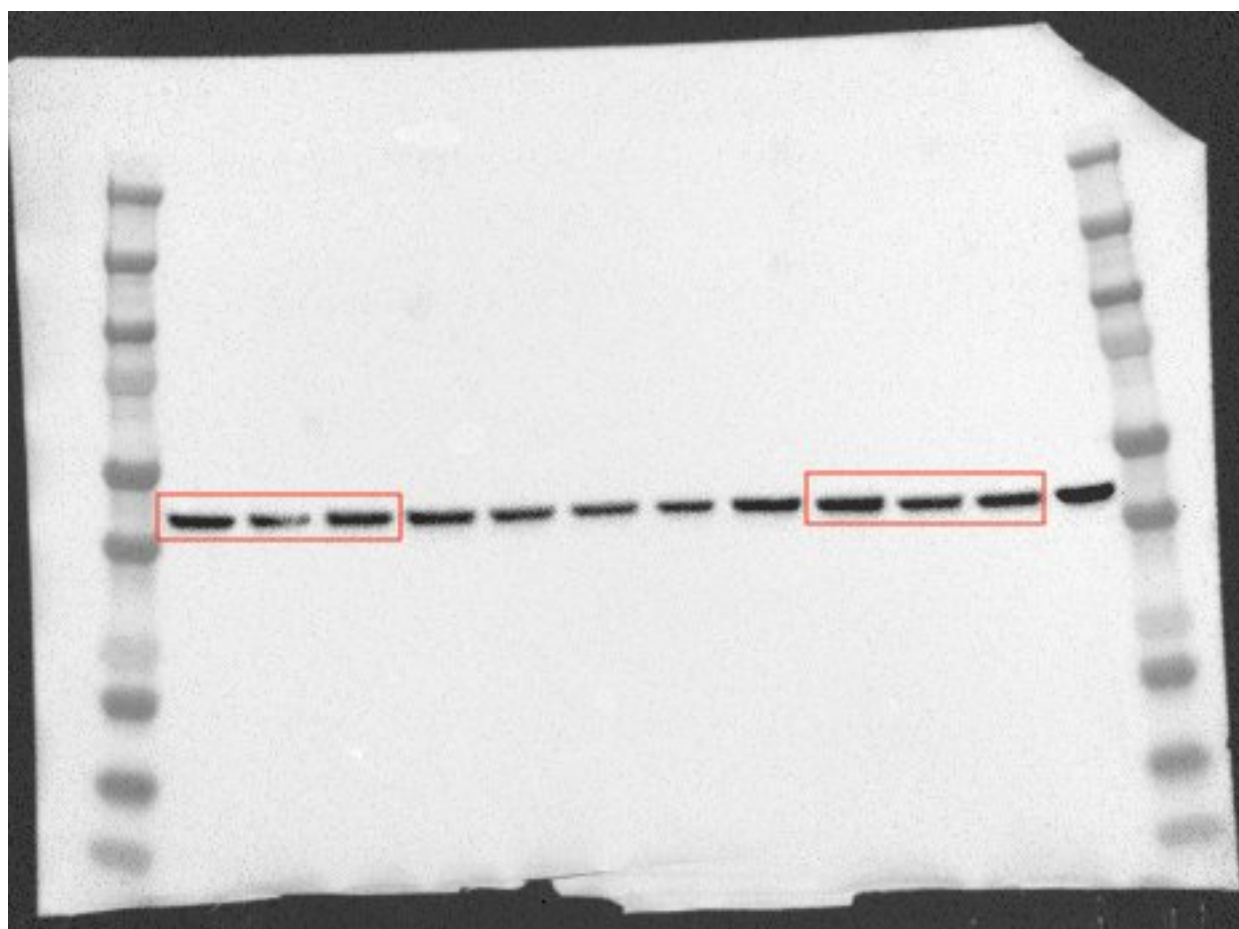

Supplement: Source Data Extended Data Fig. 8 — Unprocessed western blots. [file 43587_2022_275_MOESM26_ESM.pdf]

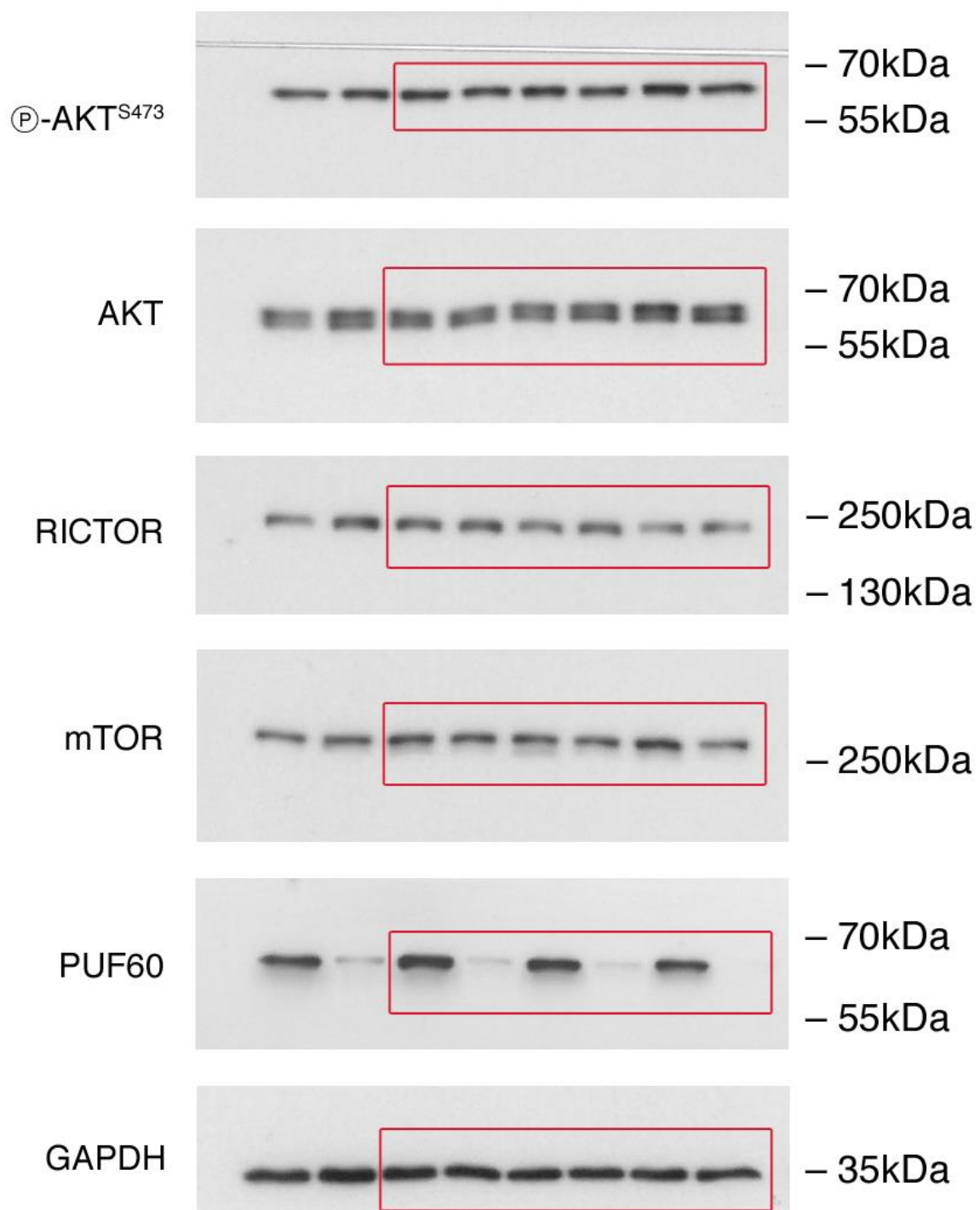

A1 B1 C1 D1 E1 F1

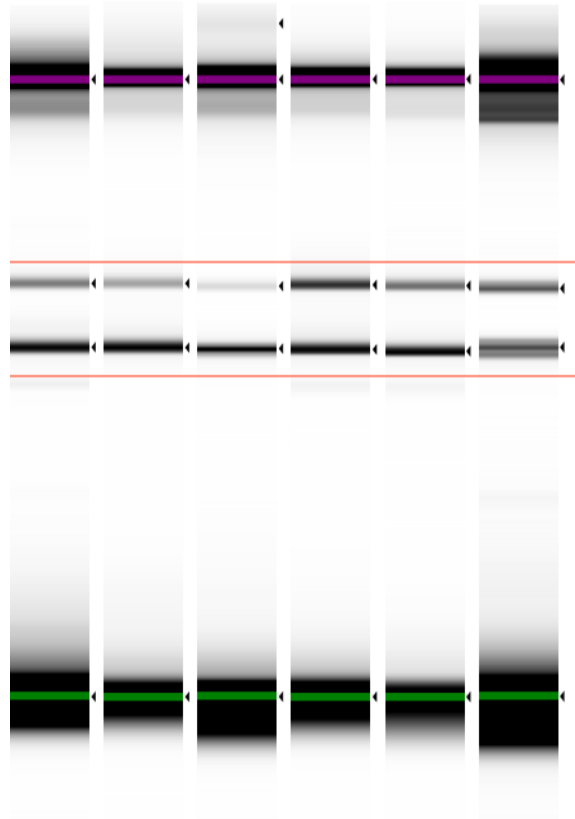

Supplement: Source Data Extended Data Fig. 9 — Unprocessed western blots and gels. [file 43587_2022_275_MOESM28_ESM.pdf]
