# Supplementary material for: Decreased spliceosome fidelity and egl-8 intron retention inhibit mTORC1 signaling to promote longevity
Source: Nat Aging. 2022 Sep 19;2(9):796–808. doi: 10.1038/s43587-022-00275-z (PMC10154236; doi:10.1038/s43587-022-00275-z)
Supplement: Source Data Fig. 5 — Unprocessed western blots. [file 43587_2022_275_MOESM12_ESM.pdf]

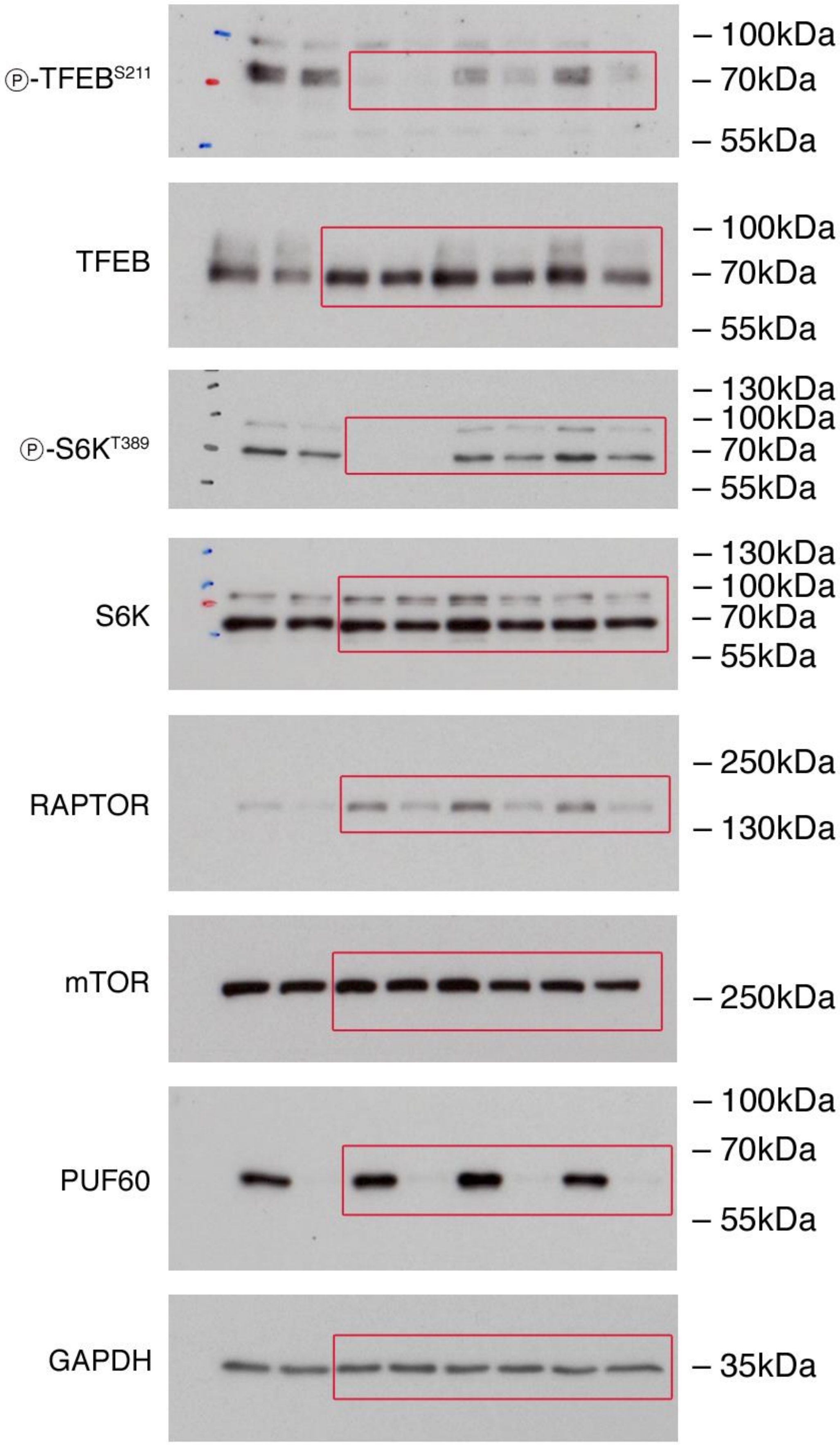

Ⓟ-TFEB S211
TFEB
Ⓟ-S6K T389
S6K
RAPTOR
mTOR
PUF60
GAPDH
100kDa
70kDa
55kDa
130kDa
250kDa
35kDa
