# Supplementary material for: Decreased spliceosome fidelity and egl-8 intron retention inhibit mTORC1 signaling to promote longevity
Source: Nat Aging. 2022 Sep 19;2(9):796–808. doi: 10.1038/s43587-022-00275-z (PMC10154236; doi:10.1038/s43587-022-00275-z)

Extended Data Fig.3a
Anti HA

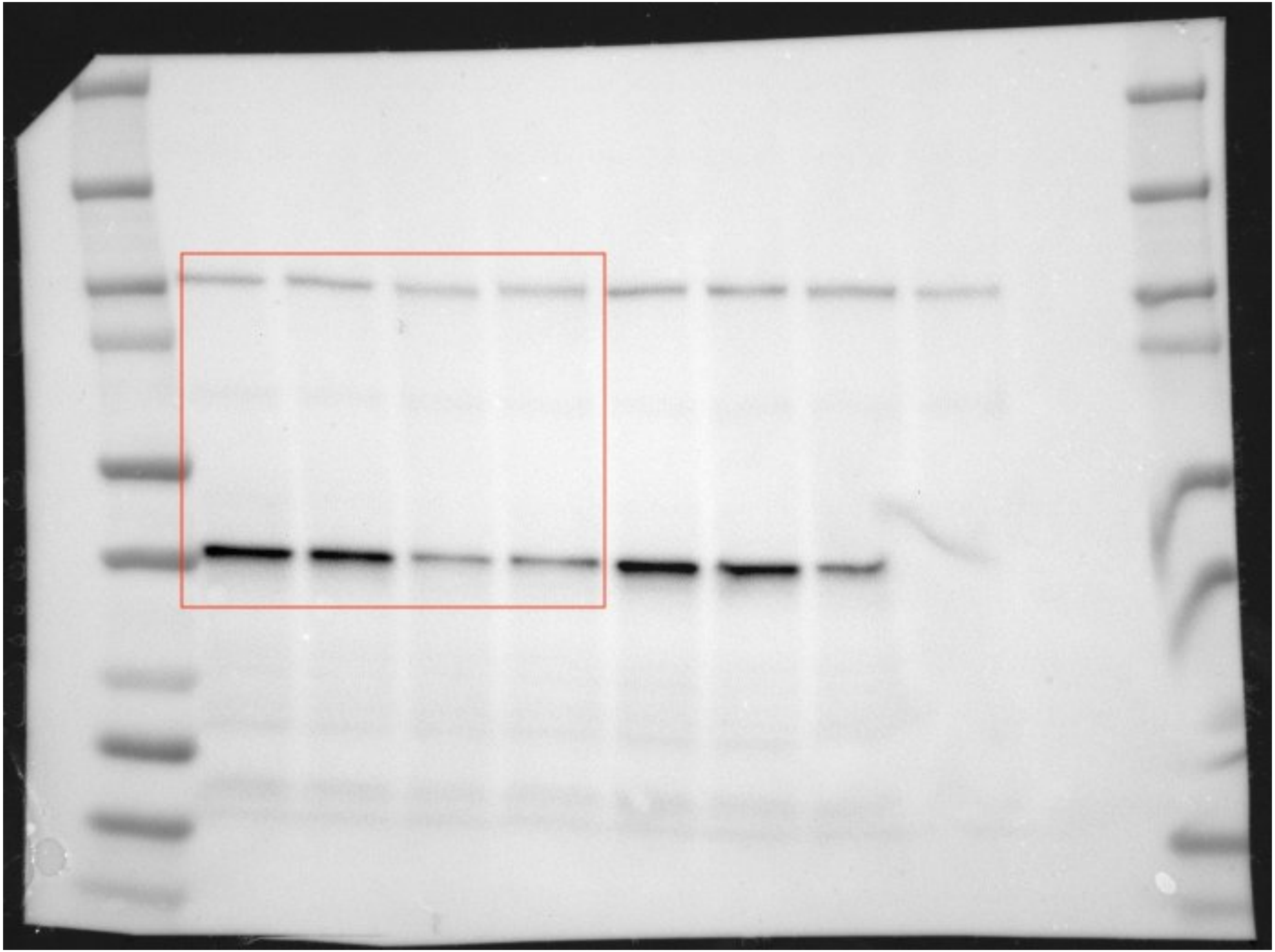

Extended Data Fig.3a
Anti Actin

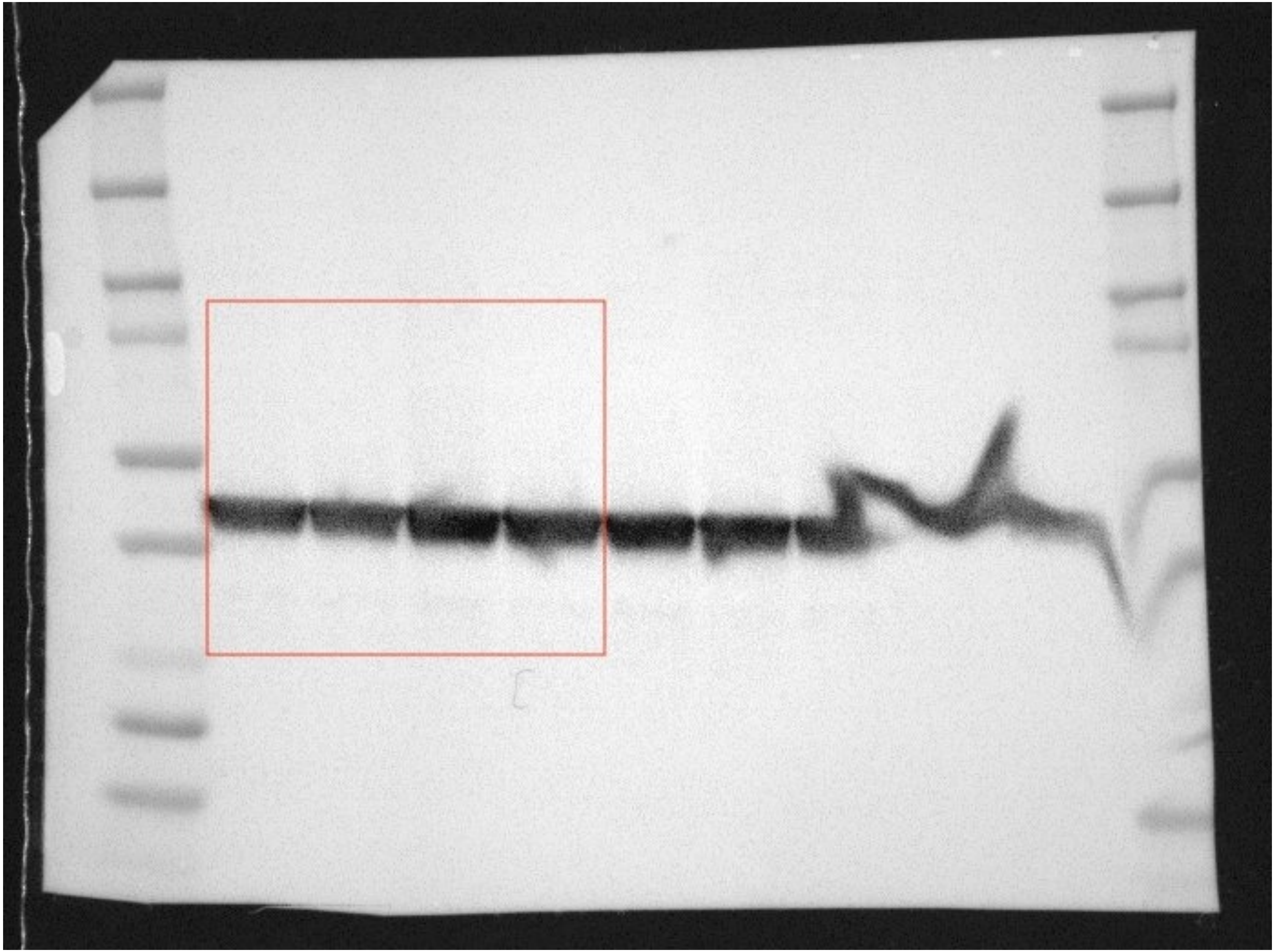

Extended Data Fig.3b

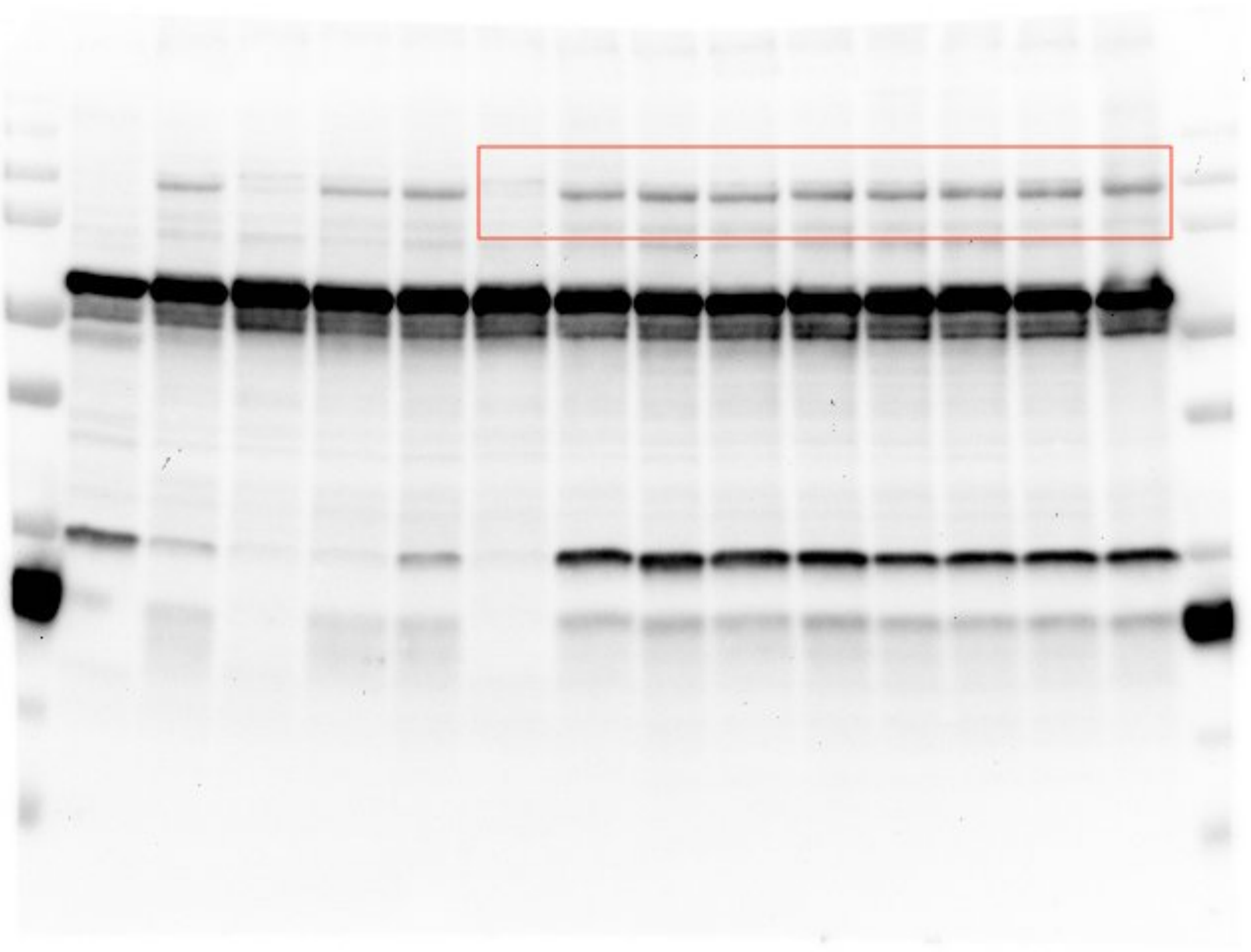

Extended Data Fig.3e
Anti HA

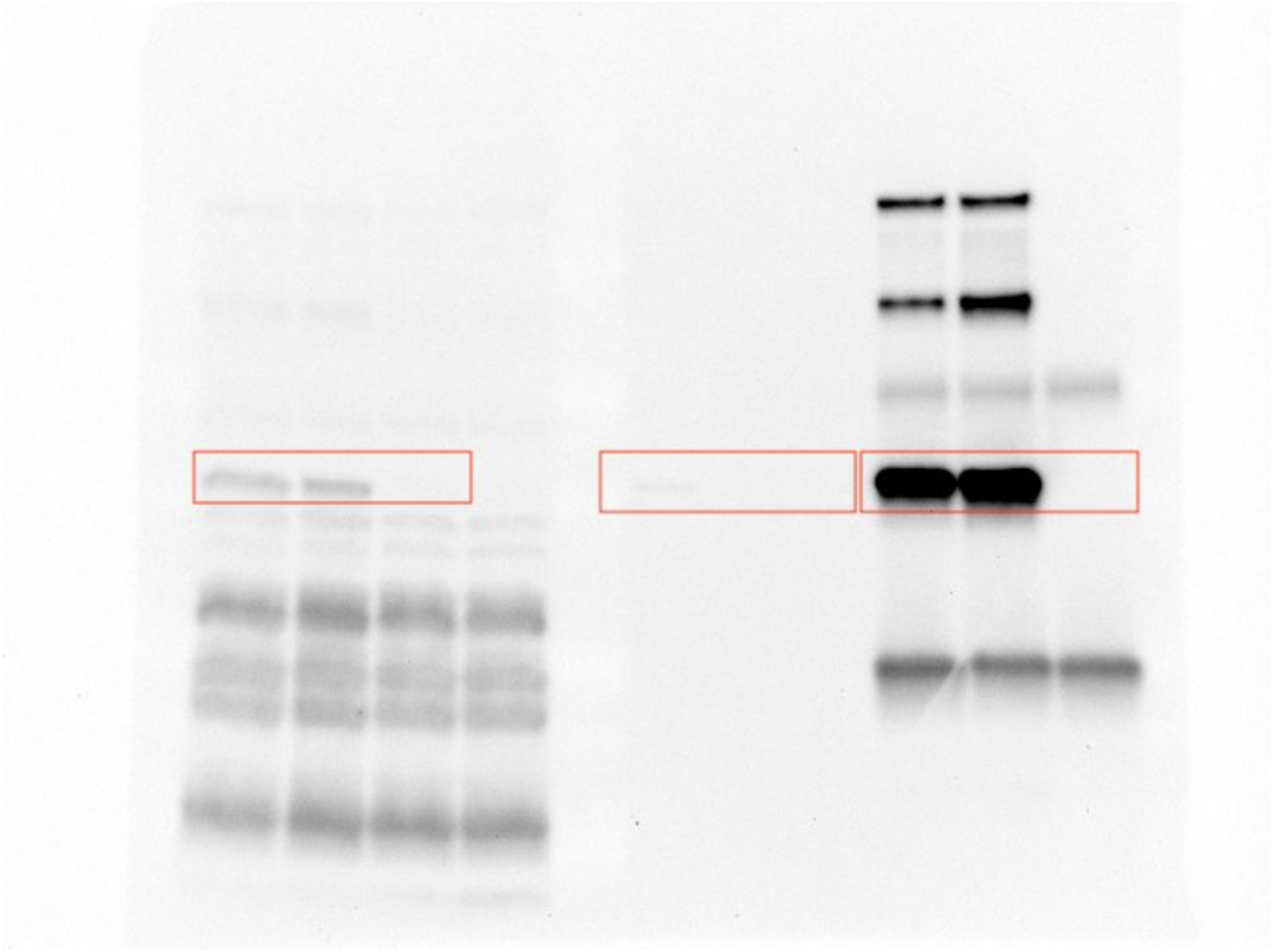

Extended Data Fig.3e
Anti mKate2

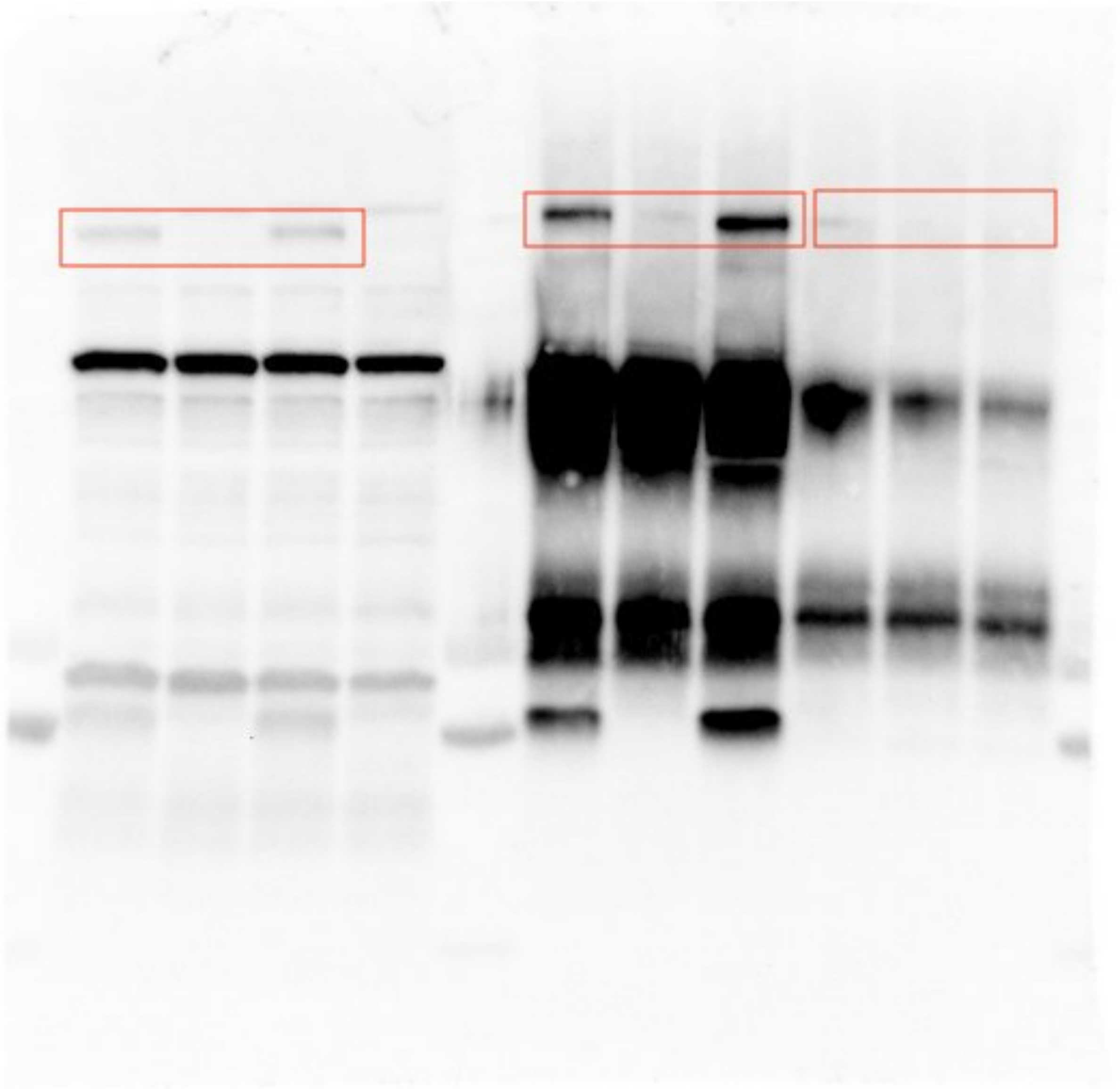

Supplement: Source Data Extended Data Fig. 3 — Unprocessed western blots. [file 43587_2022_275_MOESM17_ESM.pdf]
